# Sex-specific temporal trends in incidence and prevalence of chronic kidney disease: a Danish population-based cohort study

**DOI:** 10.1093/ckj/sfae351

**Published:** 2024-11-19

**Authors:** Anne Høy Seemann Vestergaard, Simon Kok Jensen, Uffe Heide-Jørgensen, Søren Andreas Ladefoged, Henrik Birn, Christian Fynbo Christiansen

**Affiliations:** Department of Clinical Epidemiology, Department of Clinical Medicine, Aarhus University and Aarhus University Hospital, Aarhus, Denmark; Department of Clinical Epidemiology, Department of Clinical Medicine, Aarhus University and Aarhus University Hospital, Aarhus, Denmark; Department of Clinical Epidemiology, Department of Clinical Medicine, Aarhus University and Aarhus University Hospital, Aarhus, Denmark; Department of Clinical Biochemistry, Aarhus University Hospital, Aarhus, Denmark; Department of Clinical Medicine, Aarhus University, Aarhus, Denmark; Department of Clinical Medicine, Aarhus University, Aarhus, Denmark; Department of Biomedicine, Aarhus University, Aarhus, Denmark; Department of Renal Medicine, Aarhus University Hospital, Aarhus, Denmark; Department of Clinical Epidemiology, Department of Clinical Medicine, Aarhus University and Aarhus University Hospital, Aarhus, Denmark

**Keywords:** chronic, epidemiology, health status disparities, renal insufficiency

## Abstract

**Background:**

Rates of chronic kidney disease (CKD) may change with ageing populations, rising metabolic and cardiovascular disease prevalence, increasing CKD awareness and new treatments. We examined sex-specific temporal trends in CKD incidence and prevalence from 2011 through 2021.

**Methods:**

We conducted a population-based cohort study among adults residing in the North and Central Denmark Regions (population ∼1.5 million in 2021), utilizing routinely collected individual-level laboratory data. We identified individuals with incident or prevalent CKD, using data on plasma creatinine and urine albumin–creatinine ratios from samples performed in outpatient hospital settings or primary care. We estimated annual sex-specific crude and age-standardized incidence and prevalence and tabulated clinical characteristics.

**Results:**

Throughout 2011–2021, CKD incidence and prevalence remained higher among females than males. A transient increase in the crude incidence was observed during 2011–2013, followed by a decrease from 11.8 per 1000 person-years in 2013 [95% confidence interval (CI) 11.5–12.1] to 10.7 in 2021 (95% CI 10.5–11.0) among females and from 10.9 (95% CI 10.7–11.2) to 10.6 (95% CI 10.3–10.8) among males. During 2011–2021, the crude prevalence increased among females from 85.1 per 1000 individuals (95% CI 84.4–85.8) to 99.9 (95% CI 99.2–100.6), and among males from 55.3 (95% CI 54.7–55.9) to 82.4 (95% CI 81.8–83.0). After age standardization, declines in incidence persisted, while the prevalence was stable among females, and the increase persisted among males.

**Conclusions:**

The CKD incidence and prevalence remained higher among females than males during 2011–2021. Despite a notable decline in incidence rates from 2013 onwards, the crude prevalence increased during 2011–2021.

## Graphical Abstract

**Graphical Abstract:**
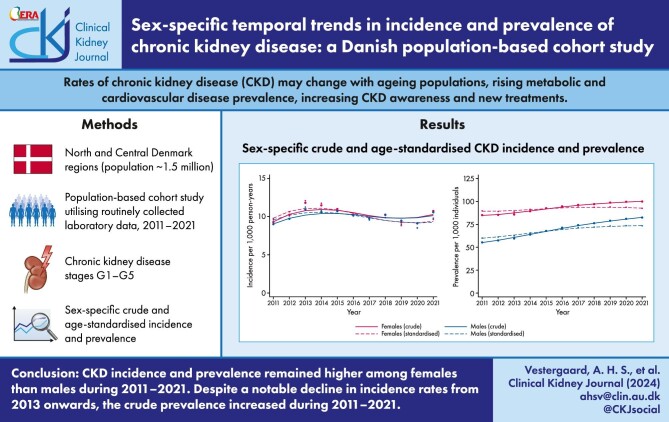

KEY LEARNING POINTS **What was known:**

- Ageing populations, rising prevalence of metabolic and cardiovascular diseases, increasing chronic kidney disease (CKD) awareness, and the introduction of new treatments aimed at preventing CKD and its progression may have affected the incidence and prevalence of CKD.
- Evidence on the sex-specific rates of CKD is primarily available from studies conducted in hospital settings.
- Understanding contemporary population-based data on sex-specific changes in CKD incidence and prevalence is crucial for designing optimal and targeted intervention strategies in screening and treatment.

**This study adds:**

- The present population-based study shows that the incidence and prevalence of CKD have remained higher among females than males from 2011 through 2021.
- From 2013 onwards, the crude and standardized incidence rates decreased for both sexes.
- From 2011 through 2021, the crude prevalence increased for both sexes, while the standardized prevalence remained stable among females and increased slightly among males.

**Potential impact:**

- Despite substantial declines in CKD incidence rates since 2013, regardless of sex, the crude prevalence of CKD increased, suggesting that the demand for healthcare services in CKD management is unlikely to have been reduced.

## INTRODUCTION

Chronic kidney disease (CKD) is defined as persistent impairment of kidney function or abnormalities in kidney structure, affecting approximately 1 in 10 adults worldwide [1, 2]. CKD markedly increases the risk of cardiovascular events, hospitalizations and premature death, leading to greater healthcare costs and having substantial impact on life expectancy and quality of life [3–7].

Considerable sex differences exist in the aetiology, detection, monitoring, progression and outcomes of CKD, and although more females than males have CKD, they are less likely to undergo treatment and receive kidney replacement therapies [8–12].

Sex-specific evidence on the rate of CKD is primarily available from studies conducted in hospital settings [9–12], despite the fact that the majority of individuals with CKD are identified, monitored and treated in primary care [13]. This issue presents challenges in understanding the sex-specific CKD rates in the general population. Furthermore, ageing populations, rising prevalence of metabolic and cardiovascular diseases, increasing CKD awareness and the introduction of new treatments aimed at preventing CKD and its progression may have affected the incidence and prevalence of CKD over the years [1, 14, 15]. Therefore, contemporary population-based data on sex-specific temporal trends in the incidence and prevalence of CKD are warranted, as they can guide intervention strategies for screening and treatment of CKD.

The aim of this study was to explore temporal trends in the incidence and prevalence of CKD among both females and males from 2011 through 2021.

## MATERIALS AND METHODS

### Study design and setting

We conducted a population-based cohort study among adults residing in the North and Central Denmark Regions (adult population ∼1.5 million in 2021) [16]. In Denmark, free healthcare services provided in hospitals or in primary care and partial reimbursement of prescription medicine are universally accessible and tax-financed through the Danish National Health Service [17].

The study was based on routinely collected individual-level information registered in population-based medical databases in Denmark. The databases can be linked using a unique identification number assigned to all Danish residents at birth or immigration [18].

The study was registered at Aarhus University (record number 2016-051-000001/812). Under Danish law, approvals from ethics committees and patient consents are not required for registry-based observational studies.

### Chronic kidney disease

We used the Clinical Laboratory Information System Research Database (LABKA) and the Register of Laboratory Results for Research (RLRR) to identify all adults (≥18 years) residing in the North and Central Denmark Regions with biochemically defined prevalent or incident CKD between 1 January 2011 and 31 December 2021. The LABKA contains laboratory data from blood and urine samples performed in hospitals and in primary care in the North and Central Denmark Regions since the 1990s. The RLRR contains laboratory data from samples performed in hospitals and primary care across the entire Danish population, with mounting coverage from 2011 onwards [19]. The laboratory data include information on analysis type, result and date and time of sampling [20, 21]. The point in time of complete reporting of laboratory data to the databases varies across municipalities, covered by different laboratories [21]. Therefore, to prevent misclassifying prevalent CKD as incident cases, as defined by laboratory data, we included municipalities once they had been covered for at least 3 years prior to inclusion (with the earliest inclusion in 2011) [21].

From the LABKA and RLRR, we retrieved information on plasma creatinine and urine albumin–creatinine ratio (uACR) measurements from samples performed in the outpatient hospital setting and in primary care. All creatinine analyses were performed using enzymatic methods standardized to the reference method of isotope-dilution mass spectrometry. For every plasma creatinine measurement, we calculated an estimated glomerular filtration rate (eGFR) using the 2009 CKD Epidemiology Collaboration creatinine equation without correction for race [22]. In accordance with the Kidney Disease: Improving Global Outcomes (KDIGO) GFR guidelines, we defined CKD by two or more eGFR values of <60 mL/min/1.73 m^2^ separated by at least 90 days and/or by two uACR measurements of >30 mg/g separated by at least 90 days [23]. Individuals with CKD in the 3 years preceding inclusion of their municipality were considered as having prevalent CKD throughout the study period. Conversely, individuals developing CKD during any given year within the study period were considered as having incident CKD in that particular year and prevalent CKD in subsequent years.

### Characteristics of individuals with chronic kidney disease

Various covariates were included to enable sex-specific characterization of individuals with incident and prevalent CKD, according to calendar period. We obtained information on sex, age and region of residence from the Danish Civil Registration System, which was established in 1968 and contains administrative data, including date of birth and death, immigration and address [18]. Additionally, we obtained data on various clinical characteristics, such as current smoking status (with a 1-year lookback period) and comorbidities, including hypertension, diabetes and cardiovascular disease (with a 10-year lookback period). These data were collected from inpatient and outpatient hospital contacts, which were recorded in the Danish National Patient Registry, a nationwide registry containing information from all hospital contacts since 1995 (Supplementary data, Table S1) [24]. Additionally, we retrieved information on prescription medicine for hypertension and diabetes redeemed at Danish pharmacies, using a 10-year lookback period, and information on prescription medicine associated with smoking, using a 1-year lookback period. These data were obtained from the Danish National Prescription Registry, which contains individual-level information on all prescriptions dispensed at Danish pharmacies since 1995 (Supplementary data, Table S1) [25]. Using this information, individuals were identified with hypertension if they had a diagnosis and/or redeemed at least two different types of antihypertensive medicine <180 days apart. Similarly, individuals with diabetes were identified from the diagnosis and/or from a single redemption of antidiabetic medicine. Smoking status was determined from surrogate markers, encompassing hospital contacts leading to diagnoses of smoking or chronic obstructive pulmonary disease and/or from redemption of prescription medicine associated with smoking (i.e. medicine for obstructive airway diseases and/or nicotine dependence).

We also collected data on utilization of prescription medicine, including paracetamol, non-steroidal anti-inflammatory drugs (including acetylsalicylic acid), opioids, sodium-glucose cotransporter-2 (SGLT-2) inhibitors, renin–angiotensin–aldosterone system (RAAS) inhibitors, diuretics and statins, from the Danish National Prescription Registry in the year leading up to the calendar year for prevalent CKD or to the date of incident CKD (Supplementary data, Table S1).

### Statistical analysis

We tabulated the distribution of the covariates separately for females and males with incident CKD and with prevalent CKD by calendar periods (2011–2013, 2014–2016, 2017–2019, 2020–2021).

We computed annual sex-specific CKD incidence rates as the number of individuals with incident CKD divided by the population at risk, that is, the entire adult population minus individuals with prevalent CKD on 1 January each year. Incidence rates were expressed as incident CKD cases per 1000 person-years, assuming one full person-year for each individual at risk on 1 January. Annual sex-specific CKD prevalence was computed as the number of individuals with prevalent CKD divided by the sum of adults living in North and Central Denmark Regions 1 January each year. Census numbers were retrieved from Statistics Denmark, and CKD prevalence was expressed per 1000 residents.

We furthermore computed age-standardized sex-specific incidence and prevalence of CKD, standardizing to the age distribution of the background population in the municipalities collectively covered by the North and Central Denmark Regions in year 2015. We plotted crude and age-standardized CKD incidence and prevalence, respectively, by calendar year.

Analyses of crude and age-standardized CKD incidence and prevalence were repeated for subgroups of individuals under the age of 60 years and 60 years or older, as well as for subgroups aged 18–49, 50–59, 60–69, 70–79 and 80 years or older. Additionally, the analyses were conducted according to CKD stages, including stage 1 (eGFR ≥90 mL/min/1.73 m^2^ and uACR >30 mg/g), stage 2 (eGFR 60–89 mL/min/1.73 m^2^ and uACR >30 mg/g), stage 3a (eGFR 45–59 mL/min/1.73 m^2^), stage 3b (eGFR 30–44 mL/min/1.73 m^2^), stage 4 (eGFR 15–29 mL/min/1.73 m^2^) and stage 5 (eGFR <15 mL/min/1.73 m^2^).

We performed supplemental analyses estimating and plotting the annual proportion of adult females and males in the general population with at least one creatinine test and with one uACR measurement, respectively, during 2011–2021.

Data were analysed on a secure remote server hosted by the Danish Health Data Authority using Stata 17 software (StataCorp 2021, Stata Statistical Software: Release 17; StataCorp LLC, College Station, TX, USA).

## RESULTS

The adult population covered by the laboratory databases in the North and Central Denmark Regions increased from 1 261 154 in 2011 to 1 537 578 in 2021. During the study period there was a gradual increase in the median age of the population {from 47 years [1st–3rd quartile (Q1–Q3) 33–62] in 2011 to 49 (33–65) years in 2021} with no major change in the sex distribution (Supplementary data, Table S2).

### Incidence of chronic kidney disease

**Figure 1:**
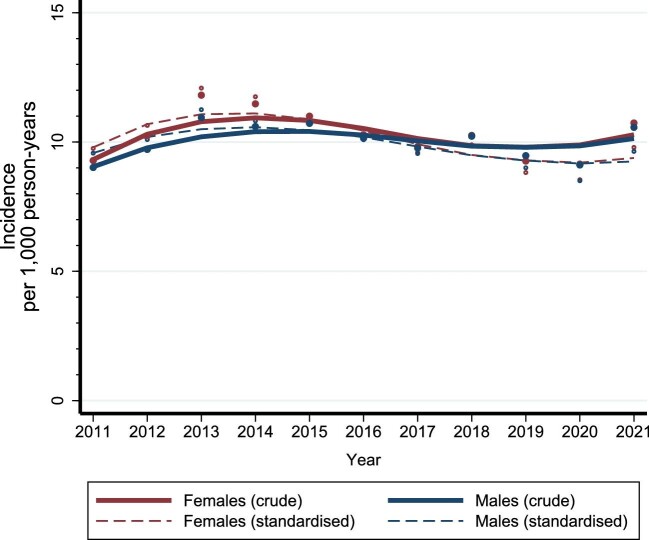
Crude and standardized incidence of CKD among females and males in Denmark between 2011 and 2021.

Throughout the study period, we observed slightly higher CKD incidence among females than among males. A transient increase in the crude incidence of CKD per 1000 person-years occurred between 2011 and 2013, rising from 9.3 [95% confidence interval (CI) 9.0–9.5] to 11.8 (95% CI 11.5–12.1) among females and from 9.0 (95% CI 8.8–9.3) to 10.9 (95% CI 10.7–11.2) among males (Fig. 1, Supplementary data, Table S3). This was followed by a steady decrease among both sexes up until 2020, and a slight increase in 2021. By 2021, the crude incidence per 1000 person-years was 10.7 (95% CI 10.5–11.0) among females and 10.6 (95% CI 10.3–10.8) among males (Fig. 1, Supplementary data, Table S3).

The sex-specific temporal trends in the standardized incidence of CKD did not differ considerably from the crude incidence (Fig. 1, Supplementary data, Table S3).

**Figure 2:**
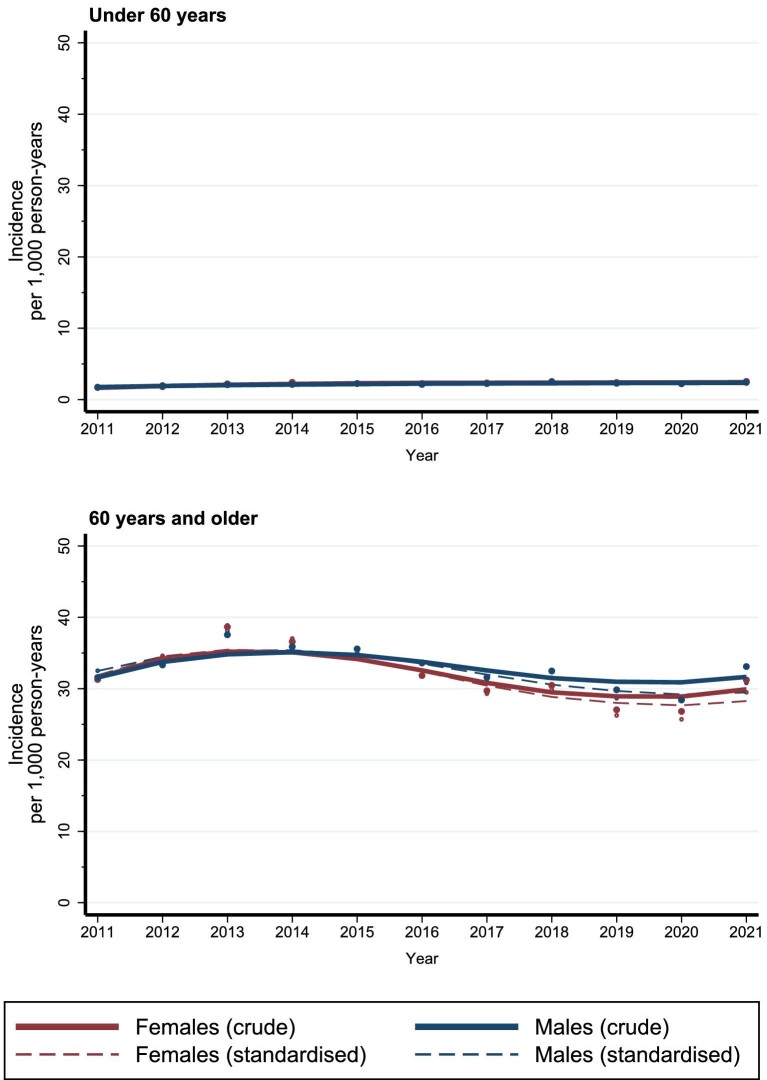
Crude and standardized incidence of CKD among females and males in Denmark, under 60 years and 60 years and older, between 2011 and 2021.

**Figure 3:**
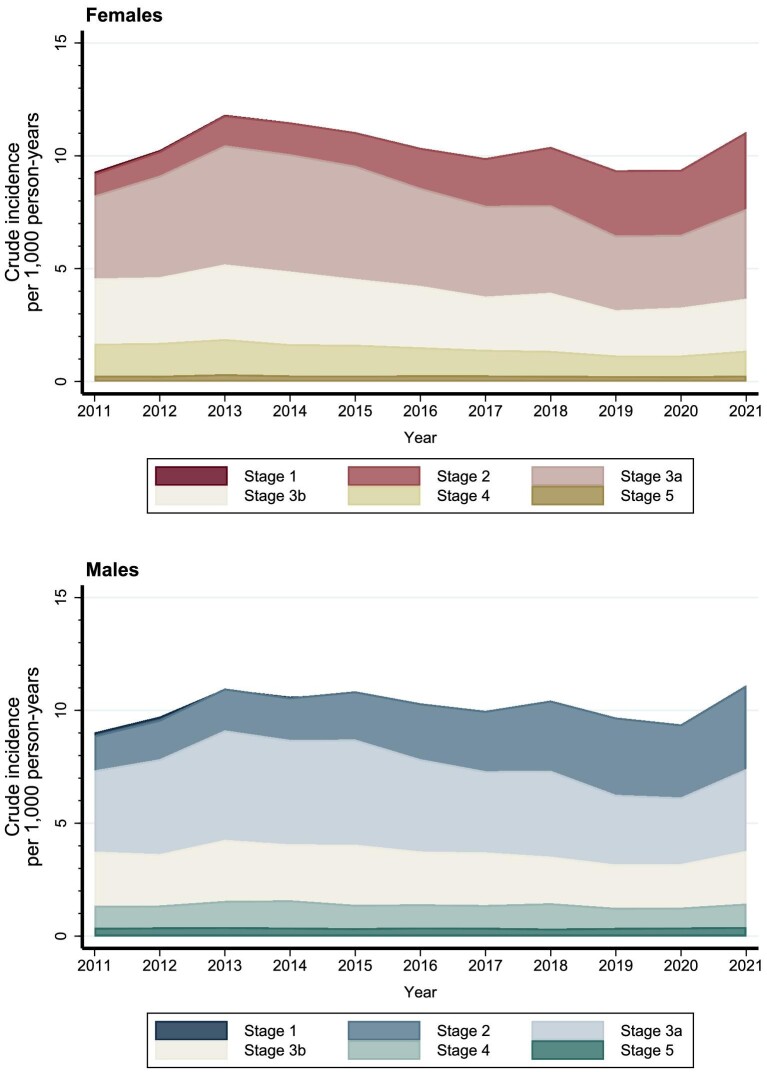
Crude incidence of CKD among females and males in Denmark according to CKD stages (1, 2, 3a, 3b, 4, 5) between 2011 and 2021.

Among both sexes, the reduction in crude and standardized incidence of CKD, from 2013 onwards, was observed primarily among individuals aged 60 years or older (Fig. 2, Supplementary data, Figs S1 and S2) and across CKD stages with the most pronounced reduction in stage 3a (Fig. 3, Supplementary data, Fig. S3). The slight increase in 2021 was mainly driven by CKD stage 2 and stage 3a (Fig. 3, Supplementary data, Fig. S3).

### Prevalence of chronic kidney disease

**Figure 4:**
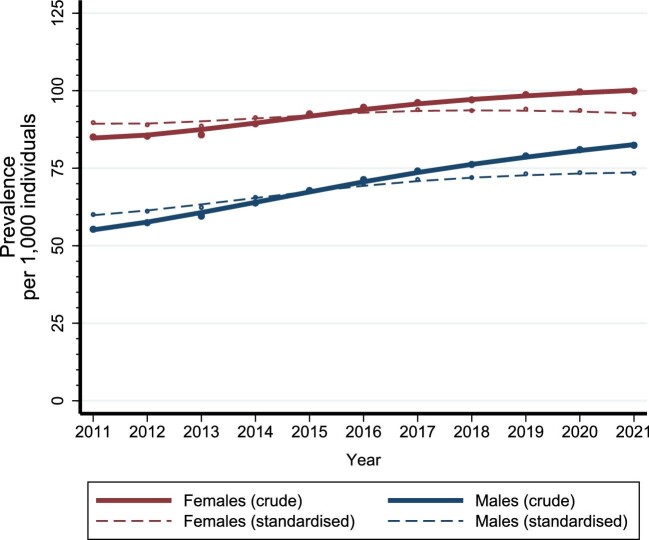
Crude and standardized prevalence of CKD among females and males in Denmark between 2011 and 2021.

Throughout the study period, the prevalence of CKD was higher among females than among males (Fig. 4, Supplementary data, Table S3).

**Figure 5:**
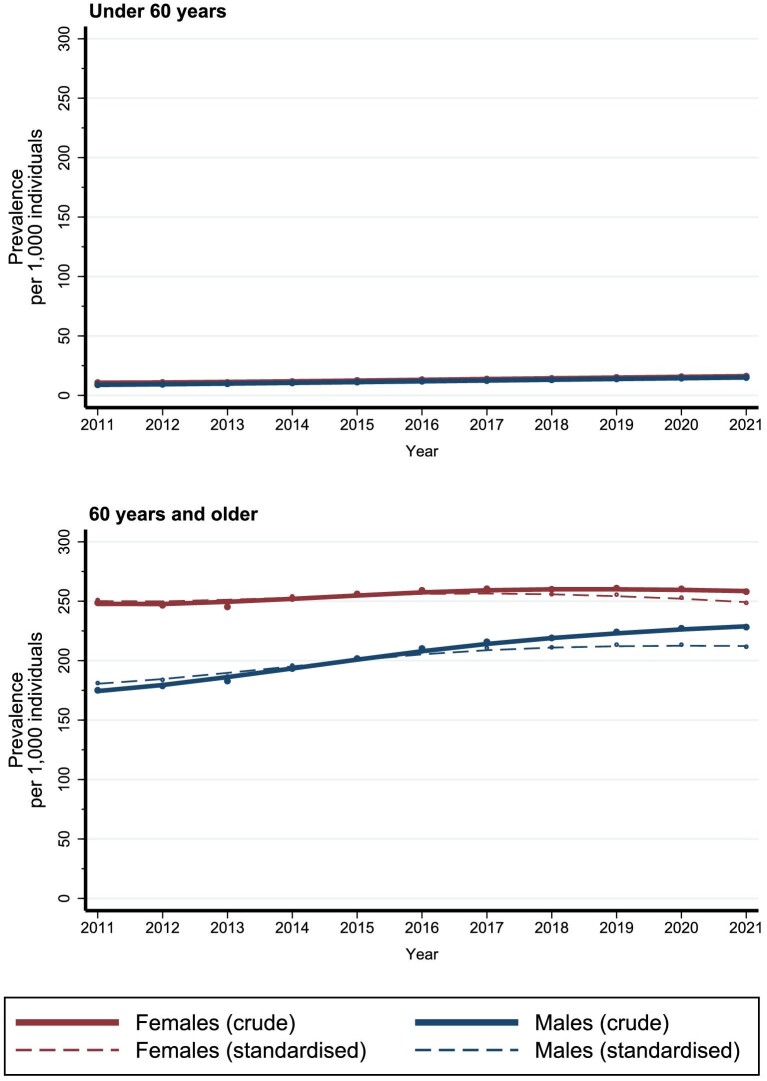
Crude and standardized incidence of CKD among females and males in Denmark, under 60 years and 60 years and older, between 2011 and 2021.

**Figure 6:**
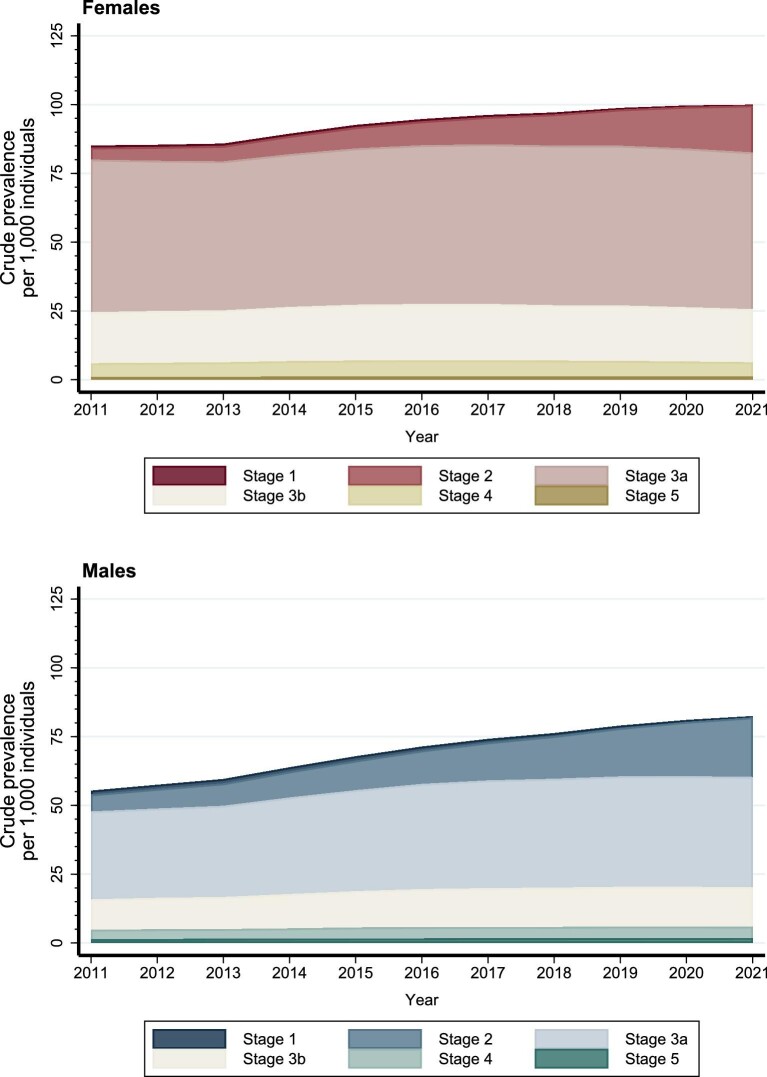
Crude prevalence of CKD among females and males in Denmark according to CKD stages (1, 2, 3a, 3b, 4, 5) between 2011 and 2021.

Among females, the crude CKD prevalence increased from 85.1 per 1000 individuals (95% CI 84.4–85.8) in 2011 to 99.9 (95% CI 99.2–100.6) in 2021 (Fig. 4, Supplementary data, Table S3). The increase was observed across age groups and primarily among females with CKD stage 1 and 2 (Figs 5 and 6, Supplementary data, Fig. S4). The standardized prevalence of CKD among females remained relatively stable, ranging from 89.7 per 1000 individuals (95% CI 89.1–90.3) in 2011 to 92.5 (95% CI 91.9–93.0) in 2021 (Fig. 4, Supplementary data, Table S3).

Among males, the crude CKD prevalence increased from 55.3 (95% CI 54.7–55.9) in 2011 to 82.4 (95% CI 81.8–83.0) in 2021 (Fig. 4, Supplementary data, Table S3). The increase was primarily observed among males aged 60 years or older and among males with CKD stage 1 and 2 (Figs 5 and 6, Supplementary data, Fig. S4). The standardized prevalence of CKD per 1000 individuals increased from 60.1 (95% CI 59.5–60.6) in 2011 to 73.4 (95% CI 72.9–73.9) in 2021 (Fig. 4, Supplementary data, Table S3).

### Characteristics of individuals with chronic kidney disease

The median age at time of incident CKD was 72 years in females [1st–3rd quartile (Q1–Q3) 64–79] and 71 years in males (Q1–Q3 64–78). In females with prevalent CKD, the median age was 77 years (Q1–Q3 69–84), while it was 75 years (Q1–Q3 67–81) in males. This remained stable during the study period (Table 1).

**Table 1:** Sex-specific characteristics of individuals with incident CKD and prevalent CKD by calendar period.

	Females				Males			
Calendar period	2011–2013	2014–2016	2017–2019	2020–2021	2011–2013	2014–2016	2017–2019	2020–2021
Incident CKD	*n* = 19 136	*n* = 21 682	*n* = 20 005	*n* = 13 751	*n* = 18 390	*n* = 21 226	*n* = 20 480	*n* = 13 817
Age, median years (Q1–Q3)	73 (65–80)	72 (65–79)	72 (63–79)	72 (64–79)	71 (64–79)	71 (64–78)	71 (63–78)	71 (63–78)
Hypertension, *n* (%)	10 529 (55.0)	10 816 (49.9)	8574 (42.9)	5300 (38.5)	9427 (51.3)	9995 (47.1)	8102 (39.6)	4844 (35.1)
Diabetes, *n* (%)	2607 (13.6)	2688 (12.4)	2054 (10.3)	1228 (8.9)	3237 (17.6)	3335 (15.7)	2608 (12.7)	1428 (10.3)
Cardiovascular disease, *n* (%)	3229 (16.9)	3314 (15.3)	2710 (13.6)	1758 (12.8)	4639 (25.3)	5083 (24.0)	4270 (20.9)	2705 (19.6)
Smoking, *n* (%)	5550 (29.0)	5573 (25.7)	3929 (19.6)	1956 (14.2)	4910 (26.7)	4983 (23.5)	3686 (18.0)	1598 (11.6)
Prescription medicine, *n* (%)								
Paracetamol	7156 (37.4)	10 926 (50.4)	9334 (46.7)	5856 (42.6)	4578 (24.9)	8059 (38.0)	7333 (35.8)	4553 (33.0)
NSAIDs (incl. ASA)	6175 (32.3)	6015 (27.7)	4331 (21.7)	2285 (16.6)	5152 (28.0)	5136 (24.2)	3660 (17.9)	1909 (13.8)
Opioids	6403 (33.5)	6664 (30.7)	5072 (25.4)	2828 (20.6)	4679 (25.4)	4981 (23.5)	3958 (19.3)	2163 (15.7)
SGLT-2 inhibitors	132 (0.7)	228 (1.1)	330 (1.7)	321 (2.3)	195 (1.1)	363 (1.7)	501 (2.5)	532 (3.9)
RAAS inhibitors	9284 (51.5)	10 110 (46.6)	8044 (40.2)	5216 (37.9)	10 021 (54.5)	10 444 (49.2)	8718 (42.6)	5320 (38.5)
Diuretics	9706 (50.7)	9313 (43.0)	6898 (34.5)	3959 (28.8)	8090 (44.0)	7926 (37.3)	6205 (30.3)	3507 (25.4)
Statins	7822 (40.9)	8075 (37.3)	6132 (30.7)	3955 (28.8)	7987 (43.4)	8533 (40.2)	6890 (33.6)	4245 (30.7)
Prevalent CKD	*n* = 170 378	*n* = 201 944	*n* = 220 575	*n* = 153 275	*n* = 113 065	*n* = 147 003	*n* = 172 563	*n* = 124 927
Age, median years (Q1–Q3)	77 (69–84)	77 (69–84)	77 (69–84)	77 (69–84)	75 (67–82)	75 (67–81)	75 (67–81)	75 (67–81)
Hypertension, *n* (%)	115 413 (67.7)	135 444 (67.1)	140 716 (63.8)	92 175 (60.1)	75 903 (67.1)	94 469 (64.3)	103 766 (60.1)	70 033 (56.1)
Diabetes, *n* (%)	31 419 (18.4)	38 036 (18.8)	40 525 (18.4)	26 909 (17.6)	28 301 (25.0)	35 969 (24.5)	39 390 (22.8)	26 592 (21.3)
Cardiovascular disease, *n* (%)	39 108 (23.0)	45 014 (22.3)	46 858 (21.2)	31 463 (20.5)	38 213 (33.8)	47 101 (32.0)	52 719 (30.6)	36 133 (28.9)
Smoking, *n* (%)	48 581 (28.5)	52 946 (26.2)	48 631 (22.1)	25 412 (16.6)	31 684 (28.0)	36 256 (24.7)	34 067 (19.7)	17 443 (14.0)
Prescription medicine, *n* (%)								
Paracetamol	75 670 (44.4)	117 925 (58.4)	131 724 (59.7)	88 253 (57.6)	32 370 (28.6)	61 409 (41.8)	75 154 (43.6)	52 364 (41.9)
NSAIDs (incl. ASA)	52 960 (31.1)	51 791 (25.7)	46 055 (20.9)	25 341 (16.3)	31 497 (27.9)	33 883 (23.1)	30 564 (17.7)	16 952 (13.6)
Opioids	62 869 (36.9)	70 130 (34.7)	68 766 (31.2)	40 860 (26.7)	29 929 (26.5)	36 088 (24.6)	37 338 (21.6)	22 510 (18.0)
SGLT-2 inhibitors	1004 (0.6)	2084 (1.0)	4010 (1.8)	4333 (2.8)	1172 (1.0)	2611 (1.8)	5309 (3.1)	6333 (5.1)
RAAS inhibitors	90 538 (53.1)	105 001 (52.0)	110 203 (50.0)	74 315 (48.5)	67 543 (59.7)	83 750 (57.0)	92 338 (53.5)	63 058 (50.5)
Diuretics	87 143 (51.2)	93 579 (46.3)	89 269 (40.5)	53 168 (34.7)	51 044 (45.2)	59 240 (40.3)	60 471 (35.0)	37 262 (29.8)
Statins	78 539 (46.1)	92 540 (45.8)	94 855 (43.0)	62 489 (40.8)	59 175 (52.3)	74 359 (50.6)	82 029 (47.5)	55 474 (44.4)

ASA, acetylsalicylic acid; NSAIDs, non-steroidal anti-inflammatory drugs.

Overall, at time of incident CKD, 11.5% of females and 14.4% of males had diabetes and 47.2% of females and 43.8% of males had hypertension. Similarly, 18.4% of females and 23.4% of males with prevalent CKD had diabetes, while 64.8% of females and 61.7% of males had hypertension. The proportion of individuals with diabetes, hypertension or cardiovascular disease decreased slightly from 2011 to 2021, both among those with incident and those with prevalent CKD (Table 1).

A large proportion of individuals with incident or prevalent CKD were prescribed diuretics, statins analgesics and/or RAAS inhibitors (Table 1). The utilization of diuretics decreased slightly in the CKD population for both sexes during 2011–2021, while the use of SGLT-2 inhibitors increased although it remained low (Table 1).

### Measurements of plasma creatinine and urine albumin–creatinine ratio

A higher proportion of females compared with males had at least one plasma creatinine measurement performed in the outpatient hospital setting or in primary care (Supplementary data, Fig. S7). In contrast, similar proportions of females and males had at least one uACR measurement (Supplementary data, Fig. S9). During the 2011–2021 period, the annual proportion of females with at least one creatinine test increased steadily from 43.0% in 2011 to 52.0% in 2021 (Supplementary data, Fig. S7). Similarly, the annual proportion of males with at least one creatinine measurement increased from 36.0% in 2011 to 43.0% in 2021 (Supplementary data, Fig. S7). The annual proportion of females with at least one uACR measurement increased from 5.8% in 2011 to 14.1% in 2021, whereas this was from 6.8% to 15.2% for males. Among both females and males, this increase in the annual proportion of individuals having plasma creatinine or uACR measurements performed was observed across all age groups, most evident among individuals aged 80 years and above (Supplementary data, Figs S8 and S10).

## DISCUSSION

In this population-based cohort study, the incidence and prevalence of CKD remained higher among females than among males from 2011 through 2021. During the study period, we observed marked reductions in the crude and standardized CKD incidence among both females and males, particularly from 2013 onwards. Despite this, the crude CKD prevalence increased among both sexes, while the standardized CKD prevalence remained stable among females and increased slightly among males.

Our findings are in line with secondary findings in prior studies showing that a higher proportion of females than males develop incident CKD, and that CKD is more prevalent among females [8, 13, 26–31]. However, to our knowledge, only few other studies have examined temporal trends in the rate of CKD solely focussing on the prevalence [1, 32–34]. Similar to our findings, these studies have shown a relatively stable CKD prevalence over time but have generally not stratified by sex. Indeed, the National Health and Nutrition Examination Survey reported a fairly stable prevalence of CKD in the general population of the USA during 2001/2004 to 2017/2020, ranging from 15.5% to 16.2% among females and from 12.4% to 13.4% among males [35]. The slight increase appeared to be primarily driven by individuals in CKD stage 1. A study from the UK, using data from a national health survey, showed an overall CKD prevalence, defined by eGFR measurements, ranging from 5.7% to 5.2% during 2003–2009/2010 [34]. Similarly, a study from Norway found a stable CKD prevalence from 1995/1997 (11.3%) to 2006/2008 (11.1%), using data from a population survey study [33]. Furthermore, the Global Burden of Disease Chronic Kidney Disease Collaboration reported a stable global age-standardized prevalence, estimating a percentage change of 1.2% (95% CI –1.1 to 3.5) during 1990–2017.

The decline in crude and standardized CKD incidence found in the present study, along with relatively stable CKD prevalence observed in previous studies, and supported by the present study, is intriguing given the concurrent rise in common risk factors for CKD in the general population, including metabolic and cardiovascular diseases [1, 14, 15]. During 2011–2021, there was an increase in the proportion of individuals with at least one plasma creatinine and one uACR measurement per year across age groups, indicating an increased testing frequency, which may have facilitated the identification of individuals with CKD.

The transient increase in both the crude and standardized incidence of CKD starting from 2011 and peaking in 2013 may, at least to some extent, be attributable to changes in the Danish national guidelines for diagnosis and monitoring of diabetes in 2012 [36, 37]. These new guidelines emphasized the need for more frequent haemoglobin A1c measurements in conjunction with plasma creatinine measurements as part of regular monitoring in primary care, which may have led to the detection of more incident CKD cases during 2011–2013. The overall reduction in CKD incidence may be influenced by improvements in the treatment and control of diabetes, hypertension and other potential causes of CKD, despite the rise in the proportion of individuals undergoing at least one plasma creatinine and uACR measurement per year in the general population [38–40].

Importantly, despite declines in CKD incidence rates, crude CKD prevalence increased during the study period. This suggests that the demand for healthcare services in managing CKD has not decreased. While fewer new CKD cases are emerging possibly due to preventive measures and better treatment of risk factors, existing CKD patients may live longer, contributing to the rising prevalence. Consequently, the increasing prevalence underscores the ongoing and possibly growing need for comprehensive healthcare services to manage the disease effectively.

The higher CKD incidence and in particular CKD prevalence among females than males found in the present study may be influenced by multiple factors. These include biological reasons, such as hormonal differences, potential greater susceptibility to kidney disease and differences in body composition. Moreover, females tend to have longer life expectancies than males, which may contribute to higher CKD prevalence, and differences in lifestyle factors, such as smoking, diet and physical activity, may also play a role. Additionally, higher healthcare utilization among females could lead to more frequent CKD detection. Notably, we observed a higher annual proportion of females than males with at least one plasma creatinine measurement. The reasons for this disparity are likely multifactorial. Overall, women may exhibit a more proactive engagement in preventive healthcare, resulting in more frequent blood tests as part of routine check-ups and medical visits. Additionally, reproductive health needs such as pregnancy monitoring and fertility assessments along with hormonal changes during menopause, contribute to increased blood test frequency for females [41, 42]. This may lead to a greater chance of detecting CKD in females when compared with males.

The present study has several strengths. The study is a population-based cohort study, encompassing a diverse and unselected population within a healthcare system that offers universal access to healthcare services. This enabled the linkage of prospectively collected individual-level data between national medical databases, which are generally considered to have high completeness and validity [20, 21, 25, 43]. Also, the study focuses on both incident and prevalent CKD, unlike other studies that focused only on prevalent CKD when examining time trends.

Some limitations of the study should be acknowledged. The study relies on clinical data, which implies that individuals identified in the study had plasma creatinine or uACR measurements performed as part of routine clinical care. Nevertheless, we believe that the impact of any misclassification is minor, since individuals were identified using comprehensive data on blood and urine samples routinely performed during any consultation in outpatient hospital settings or in primary care. Furthermore, plasma creatinine measurement is a standard component of blood test panels in Denmark, and this practice has not changed considerably during the study period.

Compared with the numeric blood pressure values, the algorithms for prescription-defined and hospital-diagnosed hypertension used in the present study show high predictive values and specificity, but their low sensitivity may lead to an underestimation of the true prevalence of hypertension [44].

While the number of municipalities covered by the RLRR and LABKA increased during the study period, any potential incompleteness in the data is likely attributable to technical factors related to data reporting, rather than selective inclusion of individuals [21].

Given the setting of the present study within a universal healthcare system with equal access to healthcare services, the sex-specific trends in CKD incidence and prevalence are likely influenced by disease-related risk factors rather than disparities in access to care. Although Denmark has a largely homogeneous population, approximately 14% are immigrants or their descendants, introducing some diversity in the study population [45]. This indicates that the results could be generalizable to other settings, though differences in healthcare systems and socio-demographic contexts, including the ethnic composition, should be considered for applicability in specific settings.

In conclusion, we found that the incidence and prevalence of CKD remained higher among females than among males during 2011–2021. Despite substantial declines in crude and standardized CKD incidence from 2013 onwards, regardless of sex, the crude prevalence increased during the study period. Thus, for the time being, the reduction in CKD incidence does not seem to alleviate the demand for healthcare services associated with CKD management. Future research should focus on understanding underlying factors contributing to these disparities and explore potential strategies to address them.

## Supplementary Material

sfae351_Supplemental_Files

## Data Availability

The data supporting the findings of this study can be obtained from the Danish Health Data Authority and Statistics Denmark.
