# Supplementary material for: Sex-specific temporal trends in incidence and prevalence of chronic kidney disease: a Danish population-based cohort study
Source: Clin Kidney J. 2024 Nov 19;18(1):sfae351. doi: 10.1093/ckj/sfae351 (PMC11707384; doi:10.1093/ckj/sfae351)

**Supplementary Information**

*Intended for publication as an online data supplement.*

**Sex-specific temporal trends in incidence and prevalence of chronic kidney disease: a Danish population-based cohort study**

Anne Høy Seemann Vestergaard, Simon Kok Jensen, Søren Andreas Ladefoged, Henrik Birn, Christian Fynbo Christiansen.

**Table of contents**

**Table S1** Codes and definition used in the study.

**Table S2** Age and sex distribution of the adult Danish population in the North and Central Denmark Region covered by the Clinical Laboratory Information System Research Database (LABKA) and the Register of Laboratory Results for Research (RLRR) between 2011 and 2021.

**Table S3** Crude and age-standardised incidence and prevalence of CKD in the 2011-2021 period.

**Figure S1** Crude incidence of chronic kidney disease among females and males of different age groups in Denmark between 2011 and 2021.

**Figure S2** Standardised incidence of chronic kidney disease among females and males of different age groups in Denmark between 2011 and 2021.

**Figure S3** Standardised incidence of chronic kidney disease (CKD) among females and males in Denmark according to CKD stages (1, 2, 3a, 3b, 4, 5) between 2011 and 2021.

**Figure S4** Crude prevalence of chronic kidney disease among females and males of different age groups in Denmark between 2011 and 2021.

**Figure S5** Standardised prevalence of chronic kidney disease among females and males of different age groups in Denmark between 2011 and 2021.

**Figure S6** Standardised prevalence of chronic kidney disease (CKD) among females and males in Denmark according to CKD stages (1, 2, 3a, 3b, 4, 5) between 2011 and 2021.

**Figure S7** Annual proportion of individuals with at least one plasma creatinine measurement among females and males in Denmark between 2011 and 2021.

**Figure S8** Annual proportion of individuals with at least one plasma creatinine measurement among females and males of different age groups in Denmark between 2011 and 2021.

**Figure S9** Annual proportion of individuals with at least one urine albumin-creatinine ratio measurement among females and males in Denmark between 2011 and 2021.

**Figure S10** Annual proportion of individuals with at least one urine albumin-creatinine ratio measurement among females and males of different age groups in Denmark between 2011 and 2021.

**Table S1** Codes and definition used in the study.

| Variables | Data source/coding classification | Codes |
| --- | --- | --- |
| Hypertension | DNRP/ICD-10 | I10-I15 |
|  | NPR/ATC | C02A, C02B, C02C, C02DA, C02L, C03A, C03B, C03D, C03E, C03X, C07C, C07D, C08G, C09BA, C09DA, C09XA52, C02DB, C02DD, C02DG, C04, C05, C07, C07F, C08, C09BB, C09DB |
| Diabetes | DNRP/ICD-10 | E10-E14, O240-243, O245- 249, G632, H360, N083 |
|  | NPR/ATC | A10A, A10B |
| Cardiovascular disease (stroke, heart failure, acute coronary syndrome) | DNRP/ICD-10 | G459, I60, I61, I63, I64, I500, I501, I502, I503, I508, I509, I110, I130, I132, I420, I426, I427, I428, I429, I200, I21, I23 |
| Obesity | DNRP/ICD-10 | E65, E66, Z718B2 |
|  | DNRP/Procedure | KJDF |
|  | NPR/ATC | A08 |
| Prescription medicine | NPR/ATC | Analgesics: M01A, N02BA, N02BE, N01AH, N02A, R05DA, N02AX |
|  |  | SGLT-2 inhibitors: A10BD, A10BK |
|  |  | Diuretics (including MRA): C03AA01, C03AA03, C03AB01, C03AB03, C03AX01, C03EA, C07BA02, C07BA05, C07BA07, C07BA12, C07BA68, C07BB, C07BG, C09BA, C09DA, C09DX01, C09DX03, C03DA |
|  |  | RAAS inhibitors: C09 |
|  |  | Statins: C10AA, C10B |
| Markers of smoking | DNPR/ICD-10 | J41-J44, F17, Z716, Z720 |
|  | NPR/ATC | R03, N07BA |

Abbreviations: DNPR: Danish National Patient Registry; MRA: mineralocorticoid receptor antagonists; NPR: National Prescription Registry; SGLT-2: sodium-glucose cotransporter-2; RAAS: renin-angiotensin-aldosterone system.

**Table S2** Age and sex distribution of the adult Danish population in the North and Central Denmark Region covered by the Clinical Laboratory Information System Research Database (LABKA) and the Register of Laboratory Results for Research (RLRR) between 2011 and 2021.

|  | **Calendar year** | | | | | | | | | | |
| --- | --- | --- | --- | --- | --- | --- | --- | --- | --- | --- | --- |
|  | 2011 | 2012 | 2013 | 2014 | 2015 | 2016 | 2017 | 2018 | 2019 | 2020 | 2021 |
|  |  |  |  |  |  |  |  |  |  |  |  |
| Total, n (%) | 1,261,154 (100) | 1,303,809 (100) | 1,396,132 (100) | 1,406,680 (100) | 1,469,829 (100) | 1,483,963 (100) | 1,496,753 (100) | 1,508,738 (100) | 1,518,386 (100) | 1,527,537 (100) | 1,537,578 (100) |
| Females | 635,844 (50.4) | 656,519 (50.4) | 702,440 (50.1) | 738,059 (50.2) | 743,969 (50.1) | 749,929 (50.1) | 749,929 (50.1) | 755,654 (50.1) | 760,646 (50.1) | 765,541 (50.1) | 770,703 (50.1) |
| Males | 625,310 (49.6) | 647,290 (49.6) | 693,692 (49.7) | 699,237 (49.7) | 731,770 (49.8) | 739,994 (49.9) | 746,824 (49.9) | 753,084 (49.9) | 757,740 (49.9) | 761,996 (49.9) | 766,875 (49.9) |
| Age, median years (Q1-Q3) | 47  (33-62) | 48  (33-63) | 48  (33-63) | 48  (33-63) | 49  (33-64) | 49  (33-64) | 49  (33-64) | 49  (33-64) | 49  (33-64) | 49  (33-65) | 49  (33-65) |
| Age group (years), n (%) |  |  |  |  |  |  |  |  |  |  |  |
| 18-49 | 676,169 (53.6) | 693,839 (53.2) | 738,975 (52.9) | 739,156 (52.5) | 761,896 (51.8) | 764,145 (51.5) | 764,145 (51.1) | 765,423 (50.7) | 766,908 (50.5) | 767,992 (50.3) | 769,641 (50.1) |
| 50-59 | 211,749 (16.8) | 218,734 (16.8) | 233,497 (16.7) | 235,455 (16.7) | 248,377 (16.9) | 251,767 (17.0) | 255,774 (17.1) | 258,007 (17.1) | 258,373 (17.1) | 258,918 (17.0) | 258,718 (16.8) |
| 60-69 | 196,258 (15.6) | 205,093 (15.7) | 220,191 (15.8) | 221,673 (15.8) | 230,713 (15.7) | 229,067 (15.4) | 226,991 (15.2) | 225,487 (14.9) | 225,102 (14.8) | 225,022 (14.7) | 225,849 (14.7) |
| 70-79 | 111,190 (8.8) | 117,657 (9.0) | 130,158 (9.3) | 136,187 (9.7) | 149,624 (10.2) | 158,184 (10.7) | 166,829 (11.1) | 174,374 (11.6) | 180,337 (11.9) | 185,246 (12.1) | 189,757 (12.3) |
| 80+ | 65,788 (5.2) | 68,486 (5.3) | 73,311 (5.3) | 74,209 (5.3) | 79,219 (5.4) | 80,800 (5.4) | 83,014 (5.5) | 85,447 (5.7) | 87,666 (5.8) | 90,359 (5.9) | 93,613 (6.1) |

**Table S3** Crude and age-standardised incidence and prevalence of CKD in the 2011-2021 period.

|  | Crude CKD incidence rate per 1,000 person-years (95% CI) | Age-standardised CKD incidence rate per 1,000 person-years (95% CI) | Crude CKD prevalence per 1,000  person-years (95% CI) | Age-standardised CKD prevalence per 1,000 person-years (95% CI) |  |
| --- | --- | --- | --- | --- | --- |
| Females | | | | |  |
| Calendar year |  |  |  |  |  |
| 2011 | 9.3 (9.0; 9.5) | 9.8 (9.5; 10.0) | 85.1 (84.4; 85.8) | 89.7 (89.1; 90.3) |  |
| 2012 | 10.2 (10.0; 10.5) | 10.6 (10.4; 10.9) | 85.3 (84.7; 86.0) | 89.0 (88.4; 89.6) |  |
| 2013 | 11.8 (11.5; 12.1) | 12.1 (11.8; 12.4) | 85.8 (85.1; 86.4) | 88.6 (88.0; 89.2) |  |
| 2014 | 11.5 (11.2; 11.7) | 11.8 (11.5; 12.0) | 89.3 (88.7; 90.0) | 91.3 (90.7; 91.9) |  |
| 2015 | 11.0 (10.7; 11.3) | 11.0 (10.8; 11.2) | 92.5 (91.9; 93.2) | 92.5 (92.0; 93.1) |  |
| 2016 | 10.3 (10.0; 10.5) | 10.2 (10.0; 10.0) | 94.7 (94.0; 95.3) | 93.6 (93.0; 94.1) |  |
| 2017 | 9.8 (9.60; 10.1) | 9.6 (9.4; 9.8) | 96.2 (95.5; 96.8) | 93.9 (93.3; 94.4) |  |
| 2018 | 10.3 (10.0; 10.5) | 9.9 (9.7; 10.1) | 97.1 (96.4; 97.7) | 93.6 (93.1; 94.1) |  |
| 2019 | 9.3 (9.0; 9.5) | 8.8 (8.6; 9.0) | 98.7 (98.1; 99.4) | 94.1 (93.5; 94.6) |  |
| 2020 | 9.1 (8.9; 9.4) | 8.5 (8.3; 8.7) | 99.6 (99.0; 100.3) | 93.6 (93.1; 94.2) |  |
| 2021 | 10.7 (10.5; 11.0) | 9.8 (9.6; 10.0) | 99.9 (99.2; 100.6) | 92.5 (91.9; 93.0) |  |
| Males | | | | |  |
| Calendar year |  |  |  |  |  |
| 2011 | 9.0 (8.8; 9.3) | 9.6 (9.3; 9.8) | 55.3 (54.7; 55.9) | 60.1 (59.5; 60.6) |  |
| 2012 | 9.7 (9.5; 10.0) | 10.1 (9.8; 10.3) | 57.4 (56.9; 58.0) | 61.1 (60.6; 61.7) |  |
| 2013 | 10.9 (10.7; 11.2) | 11.3 (11.0; 11.5) | 59.5 (59.0; 60.1) | 62.3 (61.8; 62.9) |  |
| 2014 | 10.6 (10.4; 10.8) | 10.8 (10.6; 11.1) | 63.8 (63.2, 64.4) | 65.6 (65.1; 66.1) |  |
| 2015 | 10.7 (10.5; 11.0) | 10.7 (10.5; 11.0) | 67.8 (67.2; 68.4) | 67.8 (67.3; 68.3) |  |
| 2016 | 10.1 (9.9; 10.4) | 10.1 (9.9; 10.3) | 71.3 (70.7; 71.9) | 70.1 (69.6; 70.6) |  |
| 2017 | 9.8 (9.5; 10.0) | 9.6 (9.3; 9.8) | 74.1 (73.5; 74.7) | 71.4 (70.9; 71.9) |  |
| 2018 | 10.2 (10.0; 10.5) | 9.9 (9.6; 9.2) | 76.2 (75.6; 76.8) | 72.0 (71.5; 72.5) |  |
| 2019 | 9.5 (9.2; 9.7) | 9.0 (8.8; 9.2) | 79.0 (78.3; 79.6) | 73.2 (72.7; 73.7) |  |
| 2020 | 9.1 (8.9; 9.3) | 8.5 (8.3; 8.7) | 81.0 (80.4; 81.6) | 73.6 (73.1; 74.1) |  |
| 2021 | 10.6 (10.3; 10.8) | 9.6 (9.4; 9.9: | 82.4 (81.8; 83.0) | 73.4 (72.9; 73.9) |  |
| Abbreviations: CKD, chronic kidney disease. | | | | |  |

**Figure S1** Crude incidence of chronic kidney disease among females and males of different age groups in Denmark between 2011 and 2021.

~~
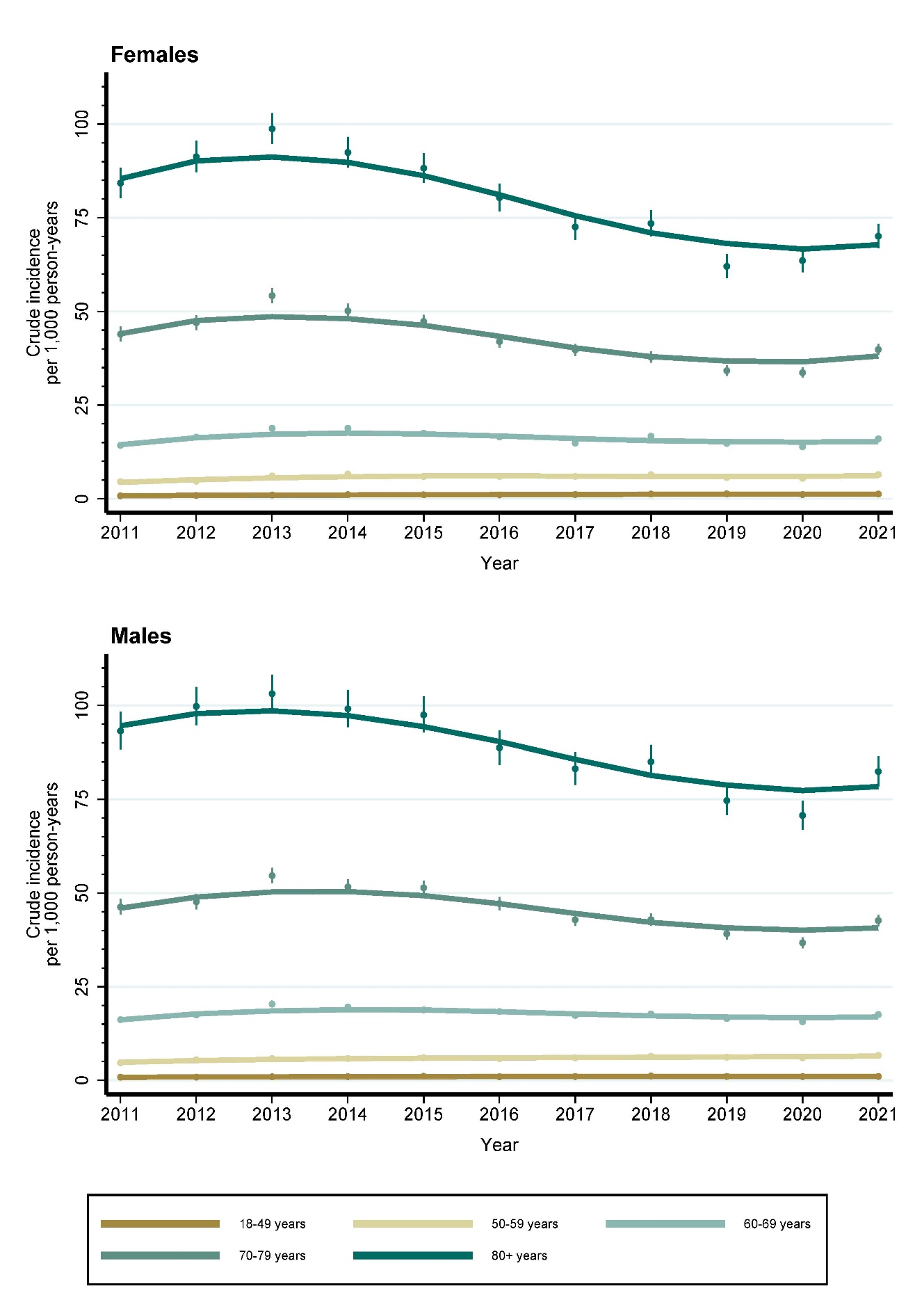
~~

**Figure S2** Standardised incidence of chronic kidney disease among females and males of different age groups in Denmark between 2011 and 2021.


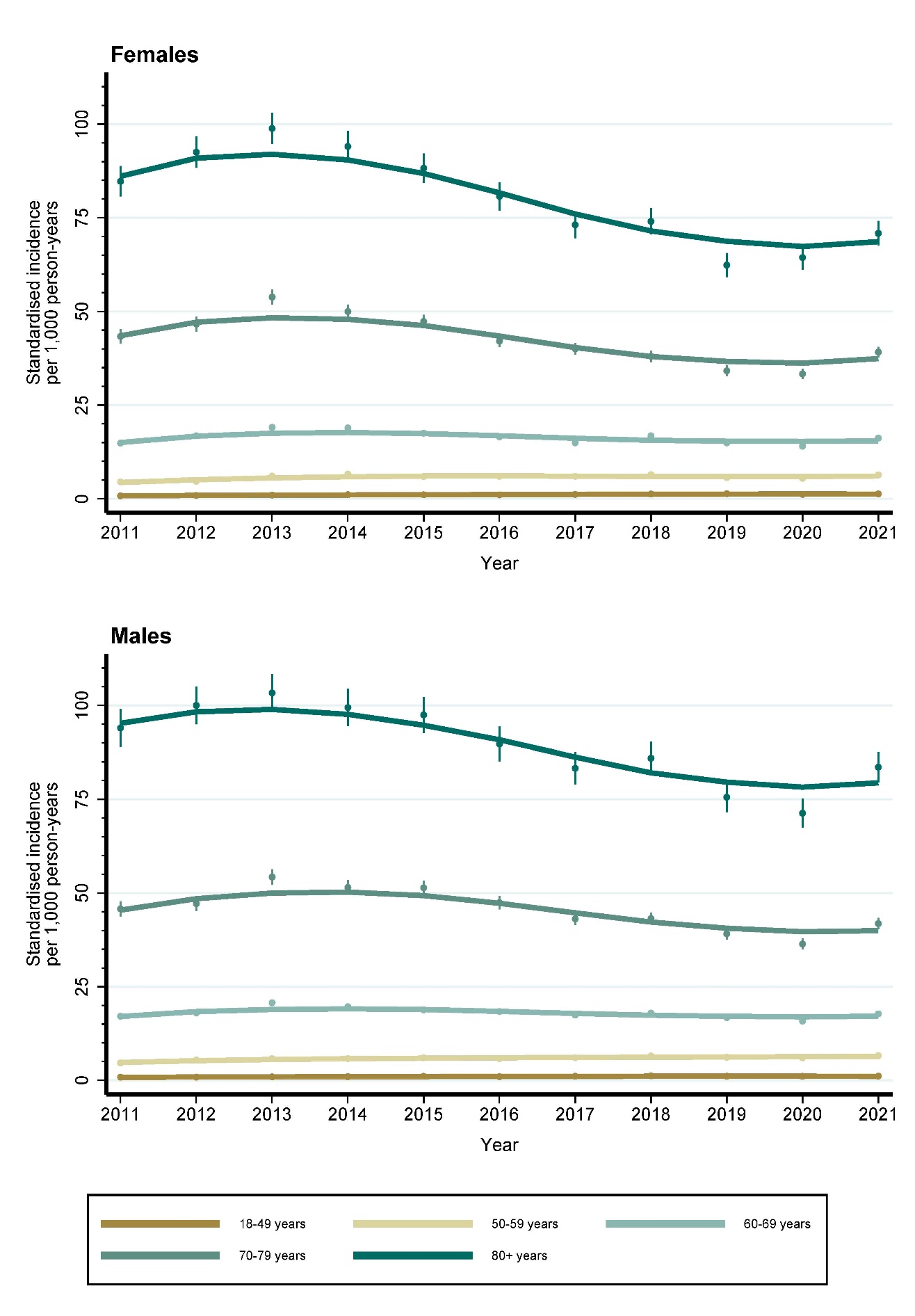


**Figure S3** Standardised incidence of chronic kidney disease (CKD) among females and males in Denmark according to CKD stages (1, 2, 3a, 3b, 4, 5) between 2011 and 2021.


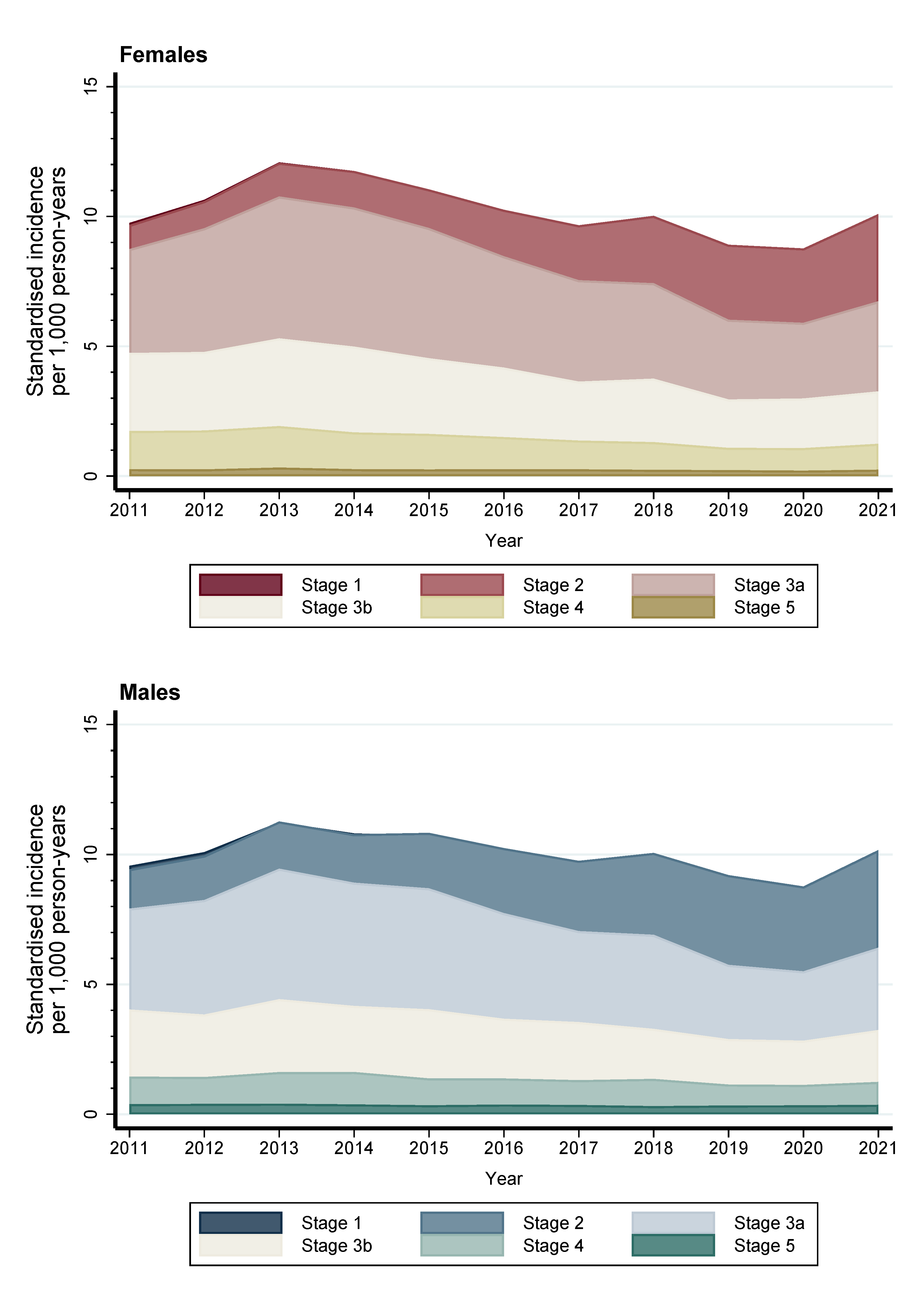


**Figure S4** Crude prevalence of chronic kidney disease among females and males of different age groups in Denmark between 2011 and 2021.


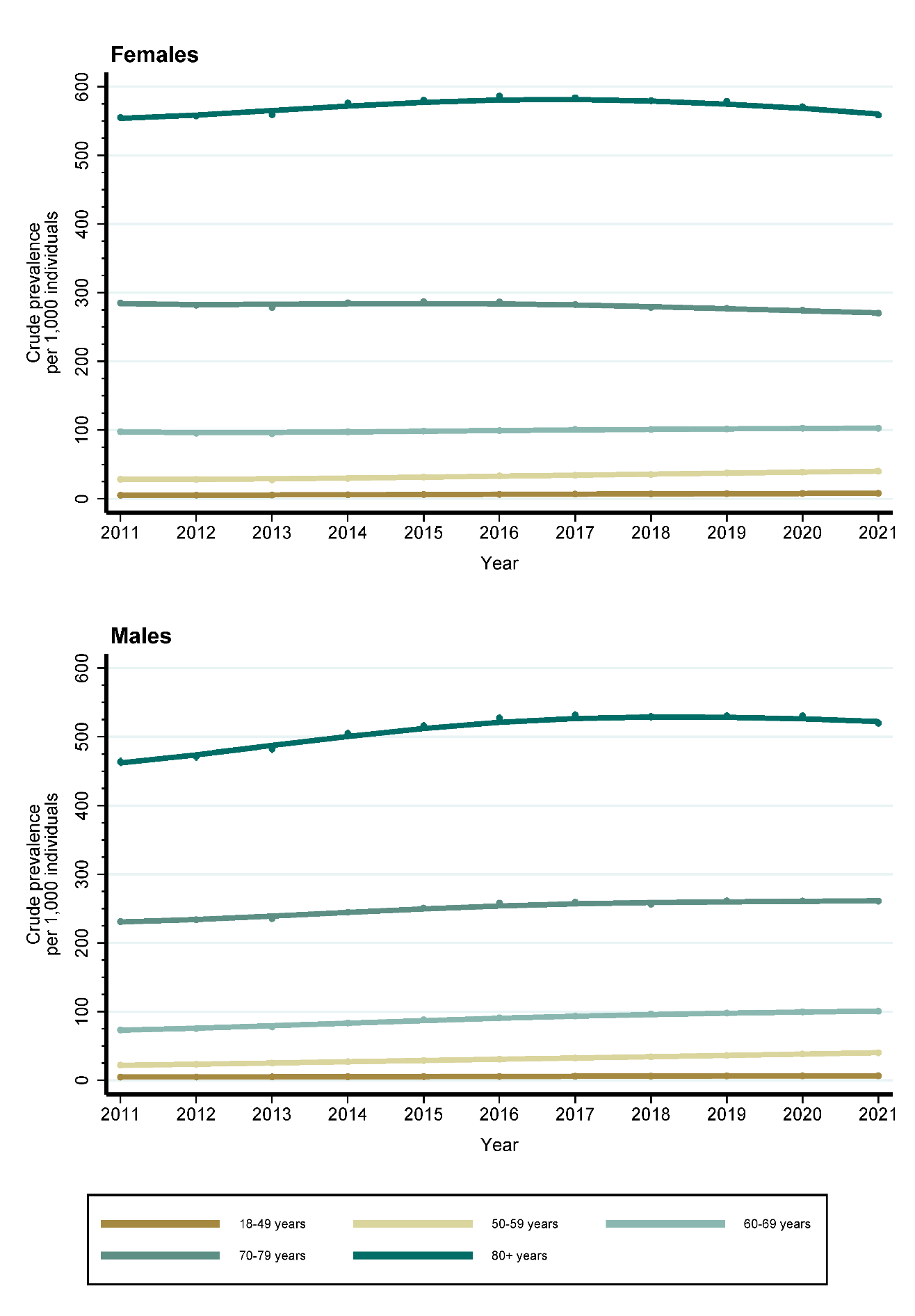


**Figure S5** Standardised prevalence of chronic kidney disease among females and males of different age groups in Denmark between 2011 and 2021.


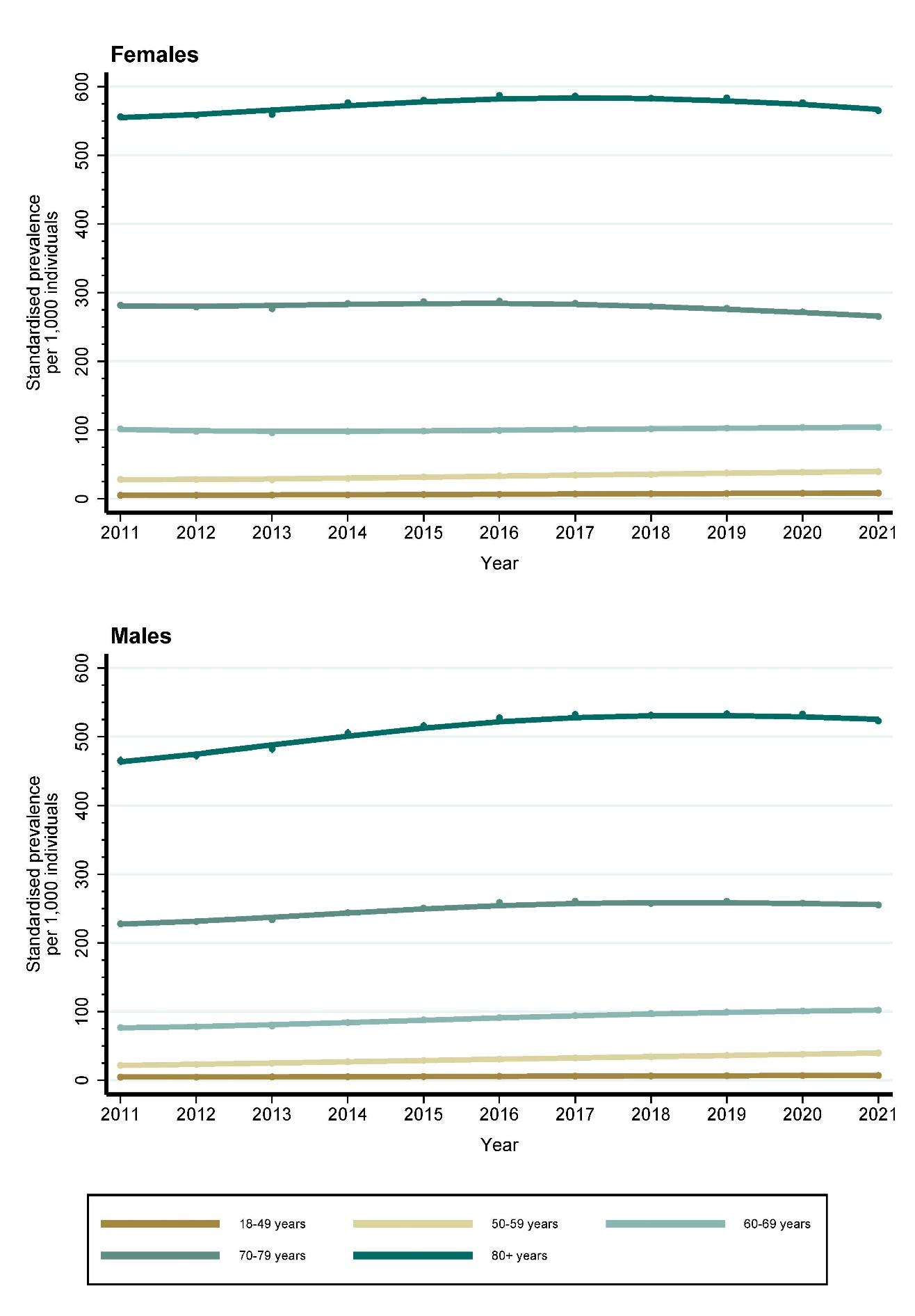


**Figure S6** Standardised prevalence of chronic kidney disease (CKD) among females and males in Denmark according to CKD stages (1, 2, 3a, 3b, 4, 5) between 2011 and 2021.

**
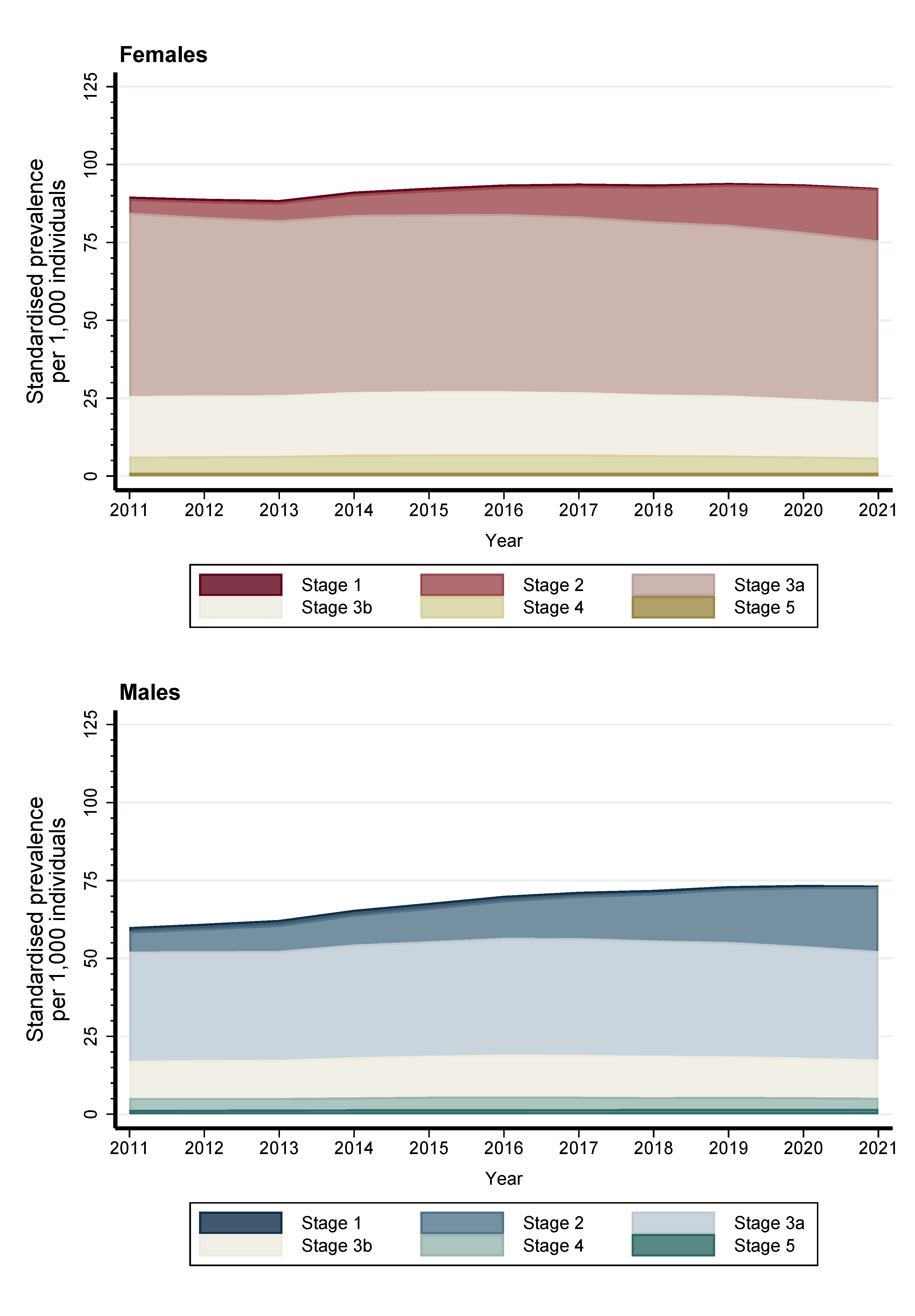
**

**Figure S7** Annual proportion of individuals with at least one plasma creatinine measurement among females and males in Denmark between 2011 and 2021.


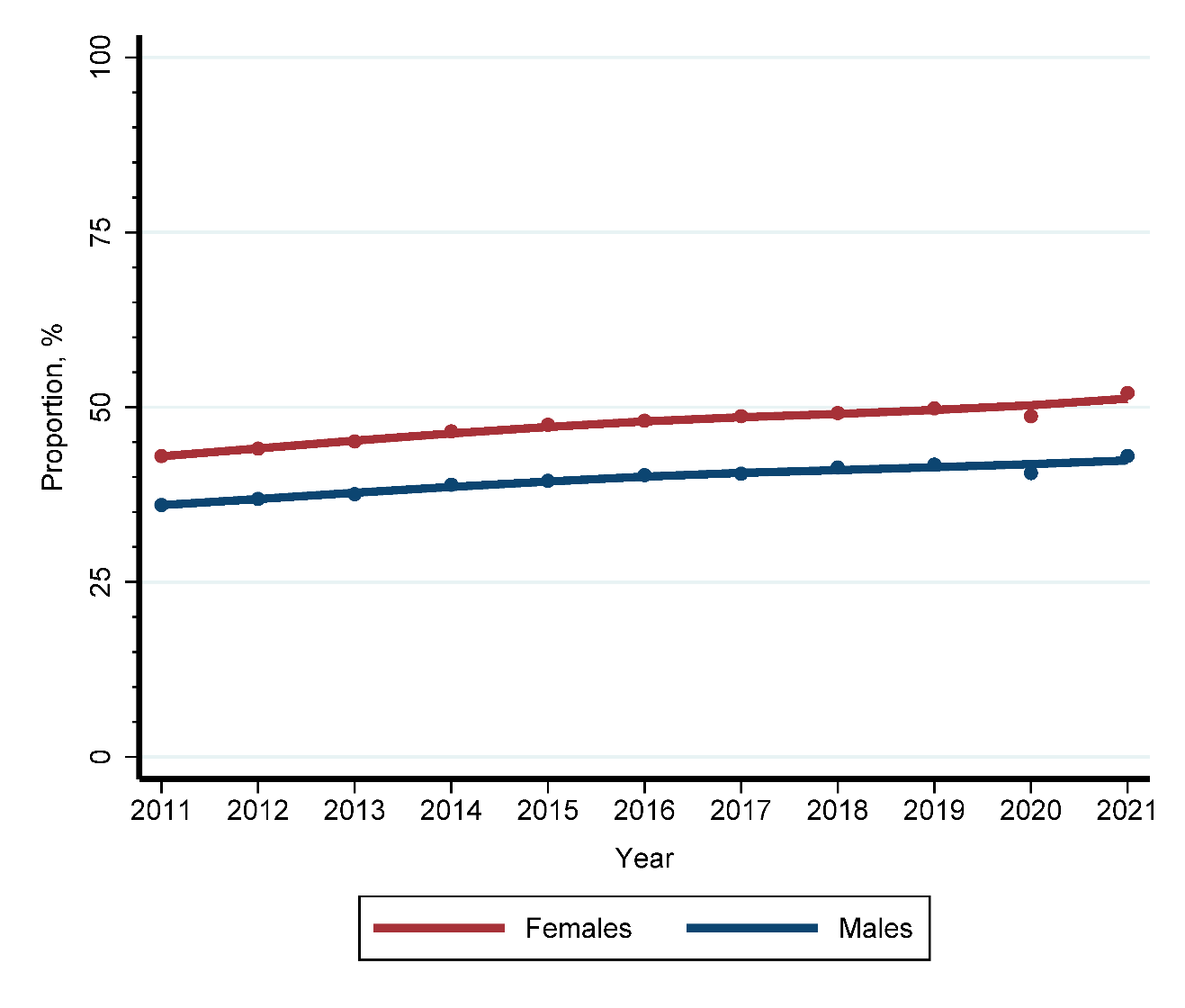


**Figure S8** Annual proportion of individuals with at least one plasma creatinine measurement among females and males of different age groups in Denmark between 2011 and 2021.


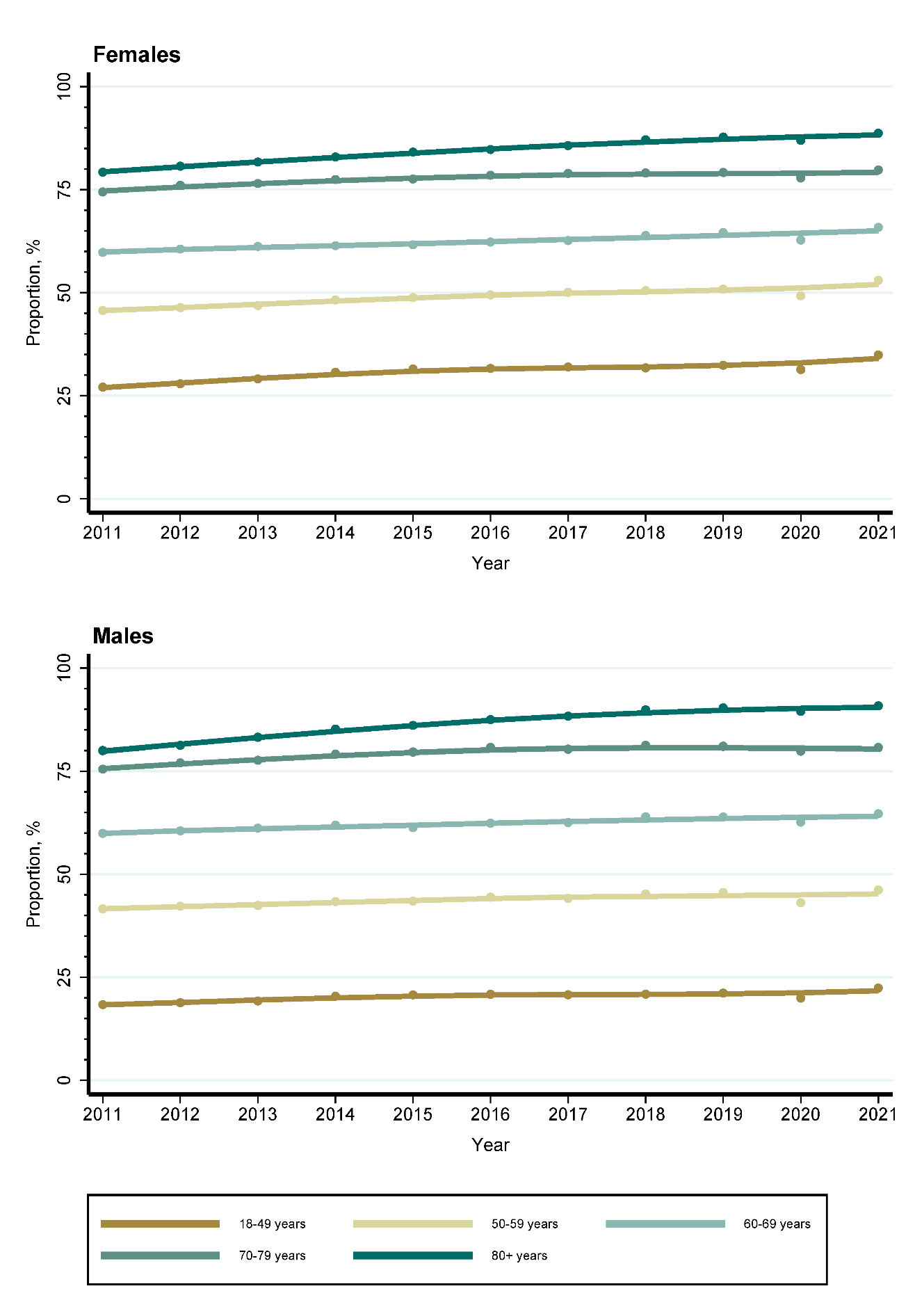


**Figure S9** Annual proportion of individuals with at least one urine albumin-creatinine ratio measurement among females and males in Denmark between 2011 and 2021.


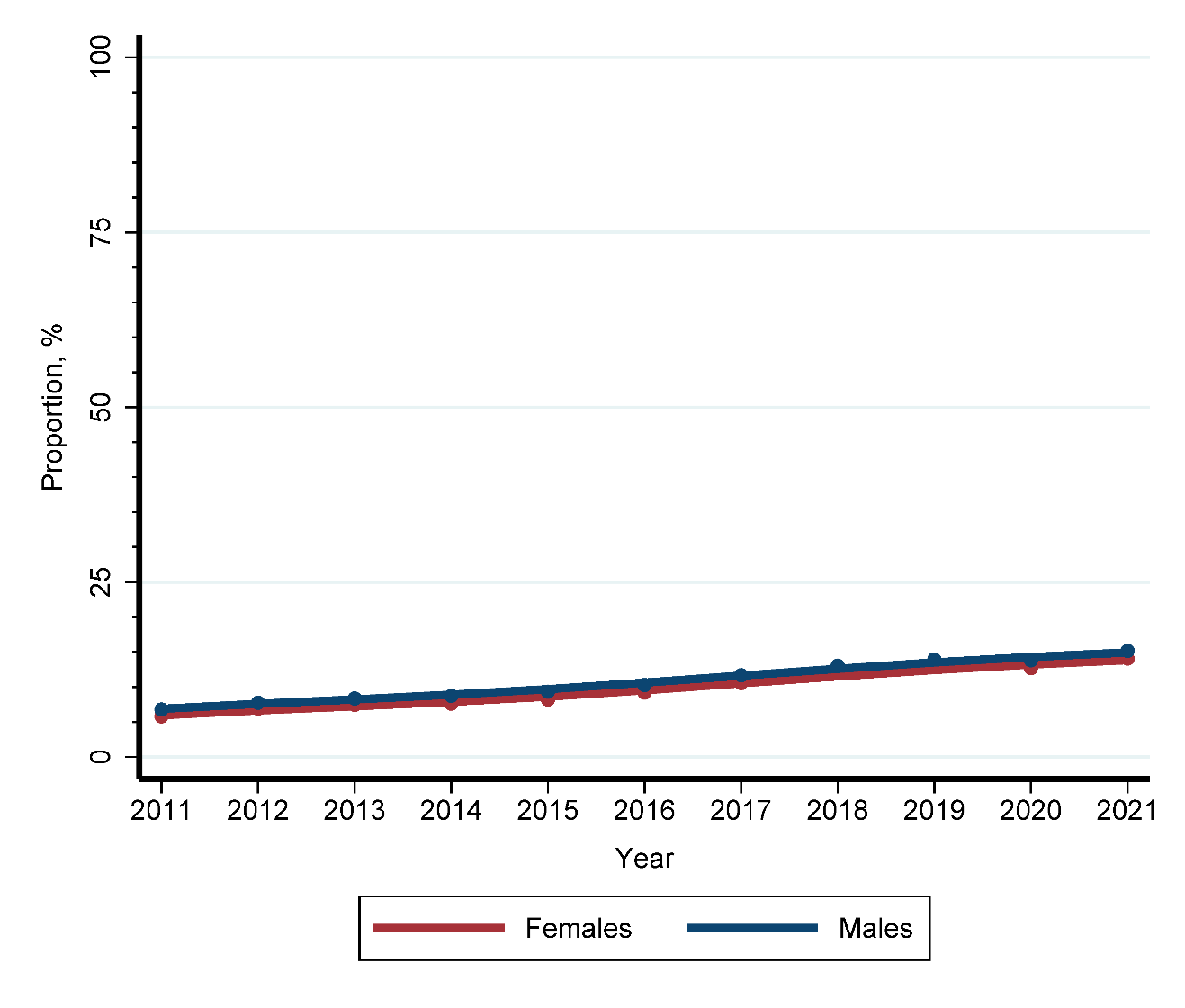


**Figure S10** Annual proportion of individuals with at least one urine albumin-creatinine ratio measurement among females and males of different age groups in Denmark between 2011 and 2021.


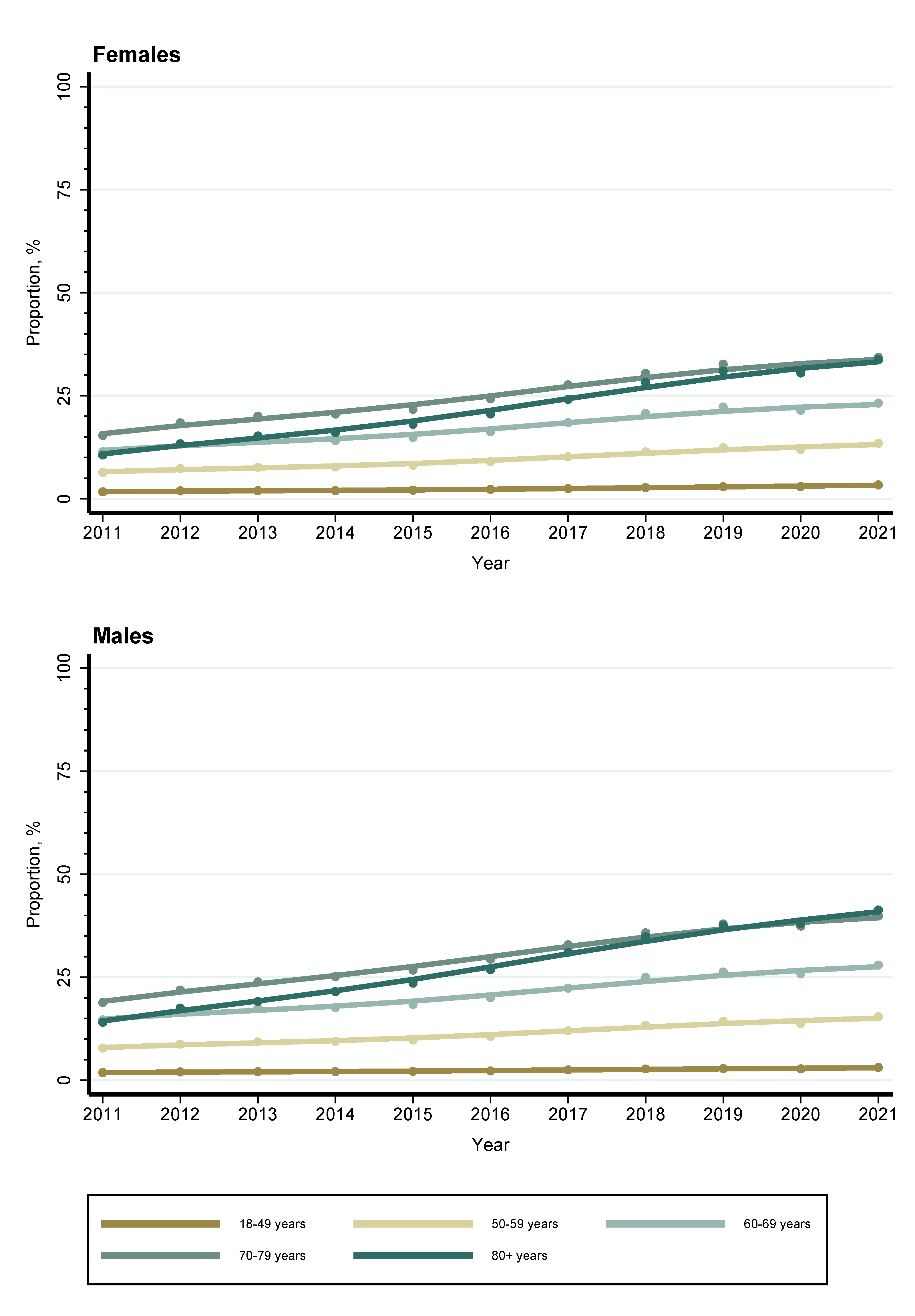

Supplement: sfae351_Supplemental_Files [file sfae351_supplemental_files.zip › Supplementary information.docx]
