# Supplementary figures and images for: Sex-specific temporal trends in incidence and prevalence of chronic kidney disease: a Danish population-based cohort study
Source: Clin Kidney J. 2024 Nov 19;18(1):sfae351. doi: 10.1093/ckj/sfae351 (PMC11707384; doi:10.1093/ckj/sfae351)

## Females

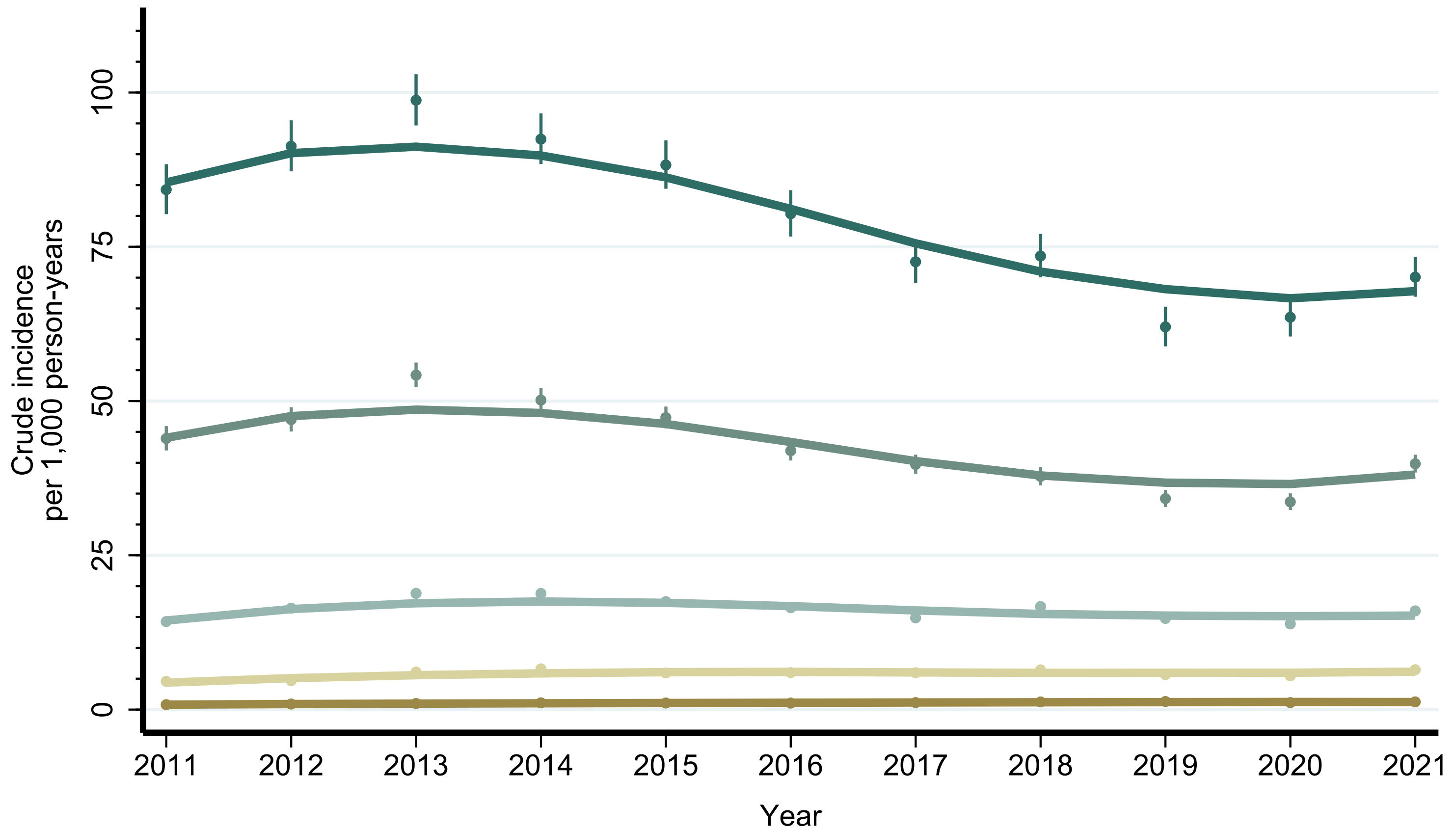

## Males

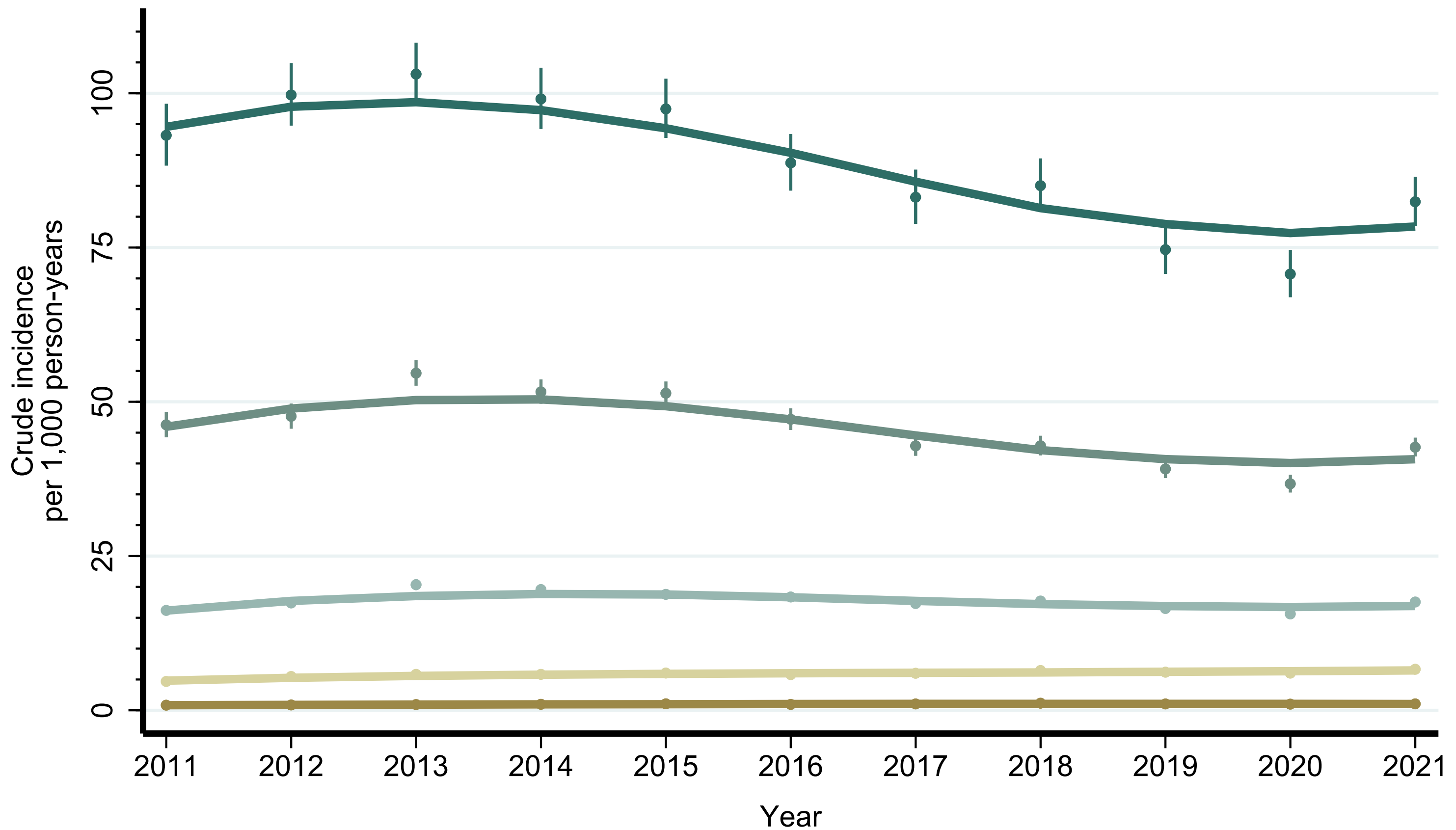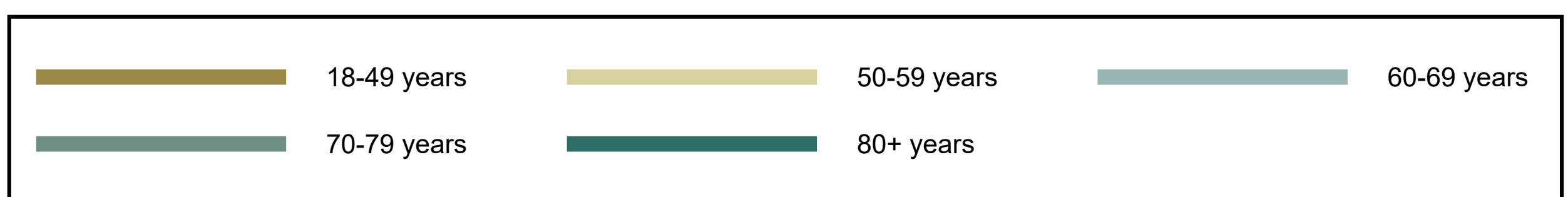

Supplement: sfae351_Supplemental_Files [file sfae351_supplemental_files.zip › Figure S1 - Crude incidence by age groups.pdf]

## Females

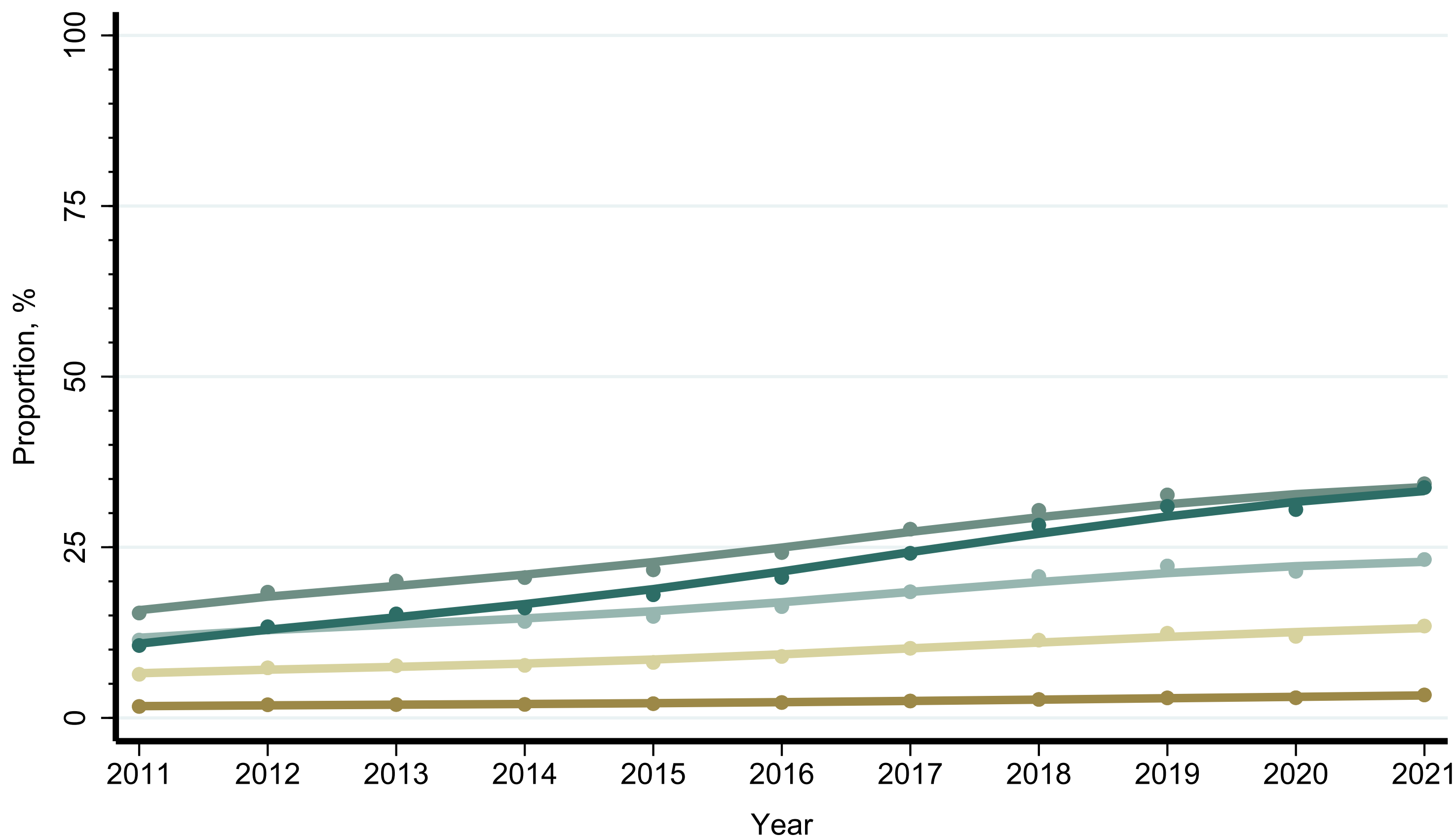

## Males

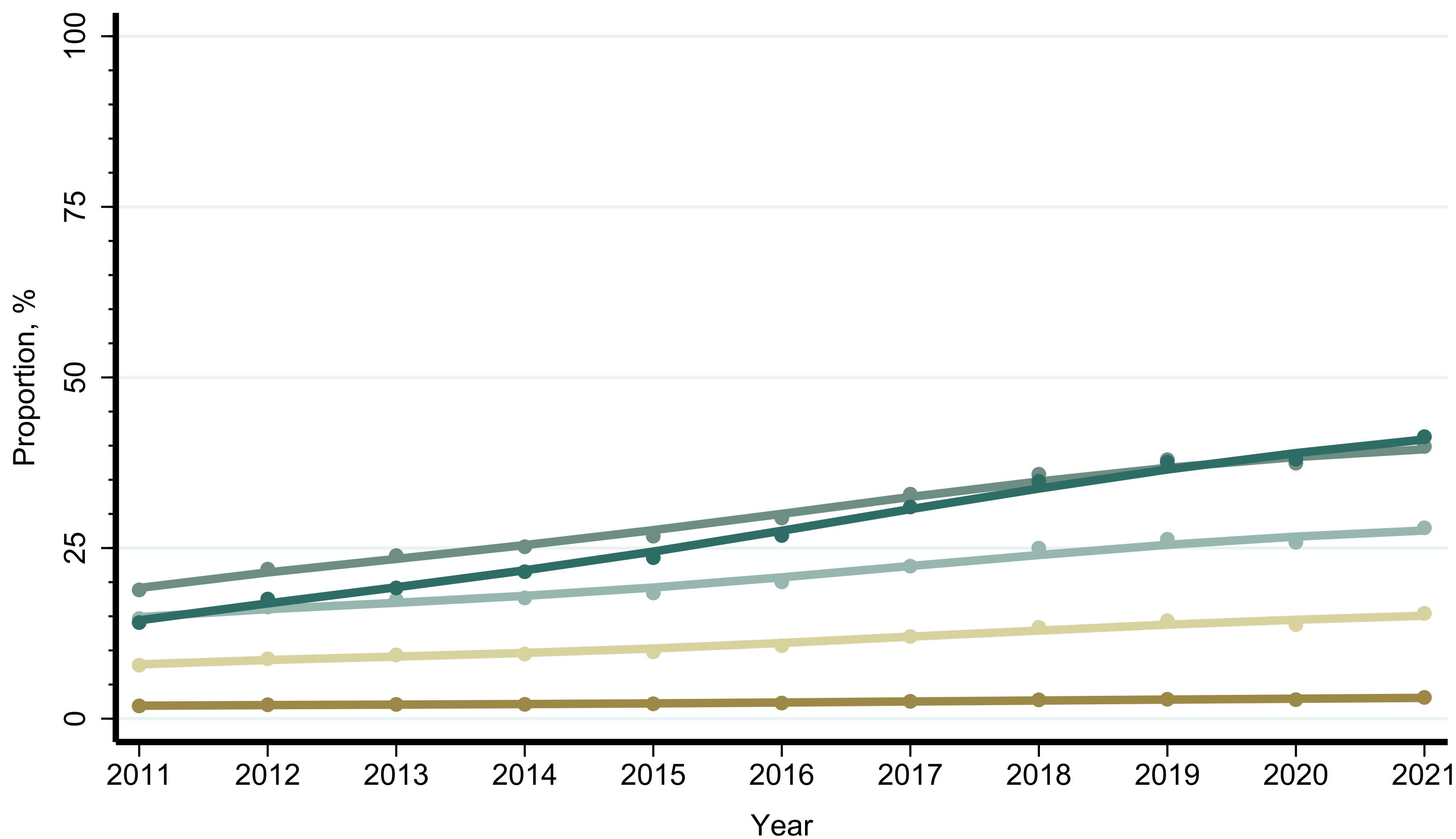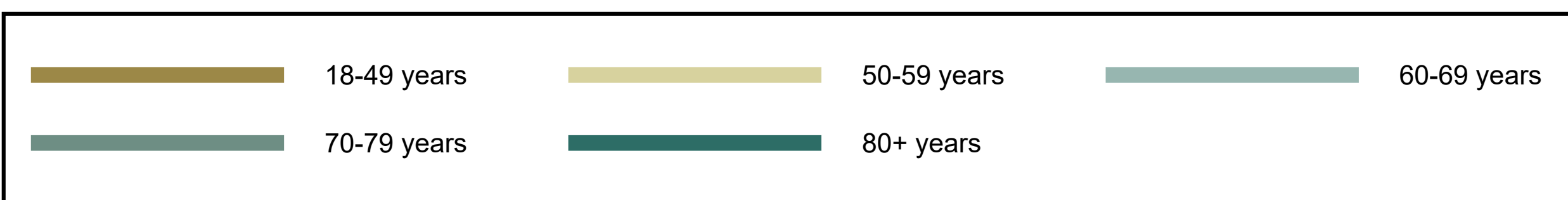

Supplement: sfae351_Supplemental_Files [file sfae351_supplemental_files.zip › Figure S10 - uACR testing by age.pdf]

## Females

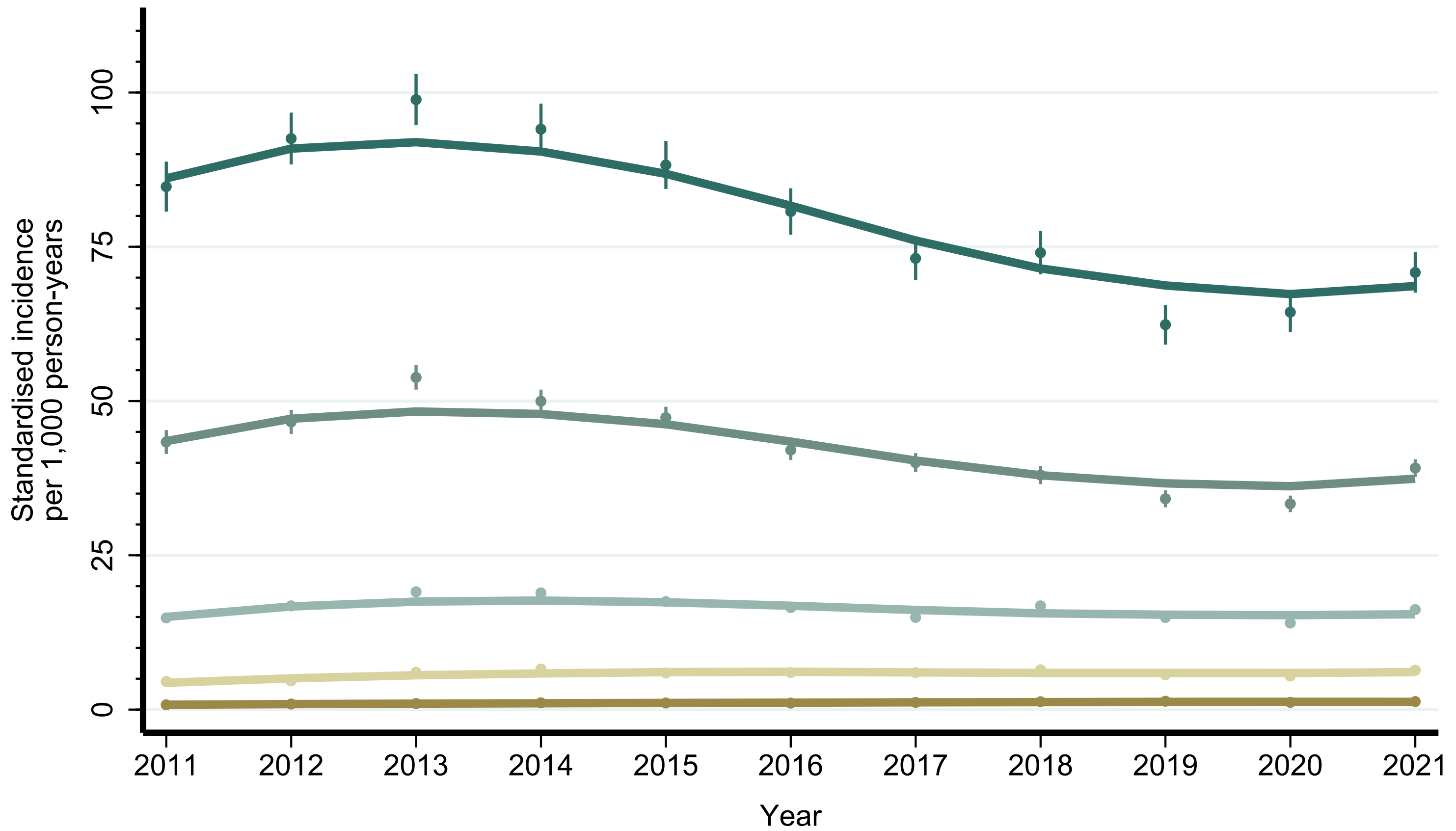

## Males

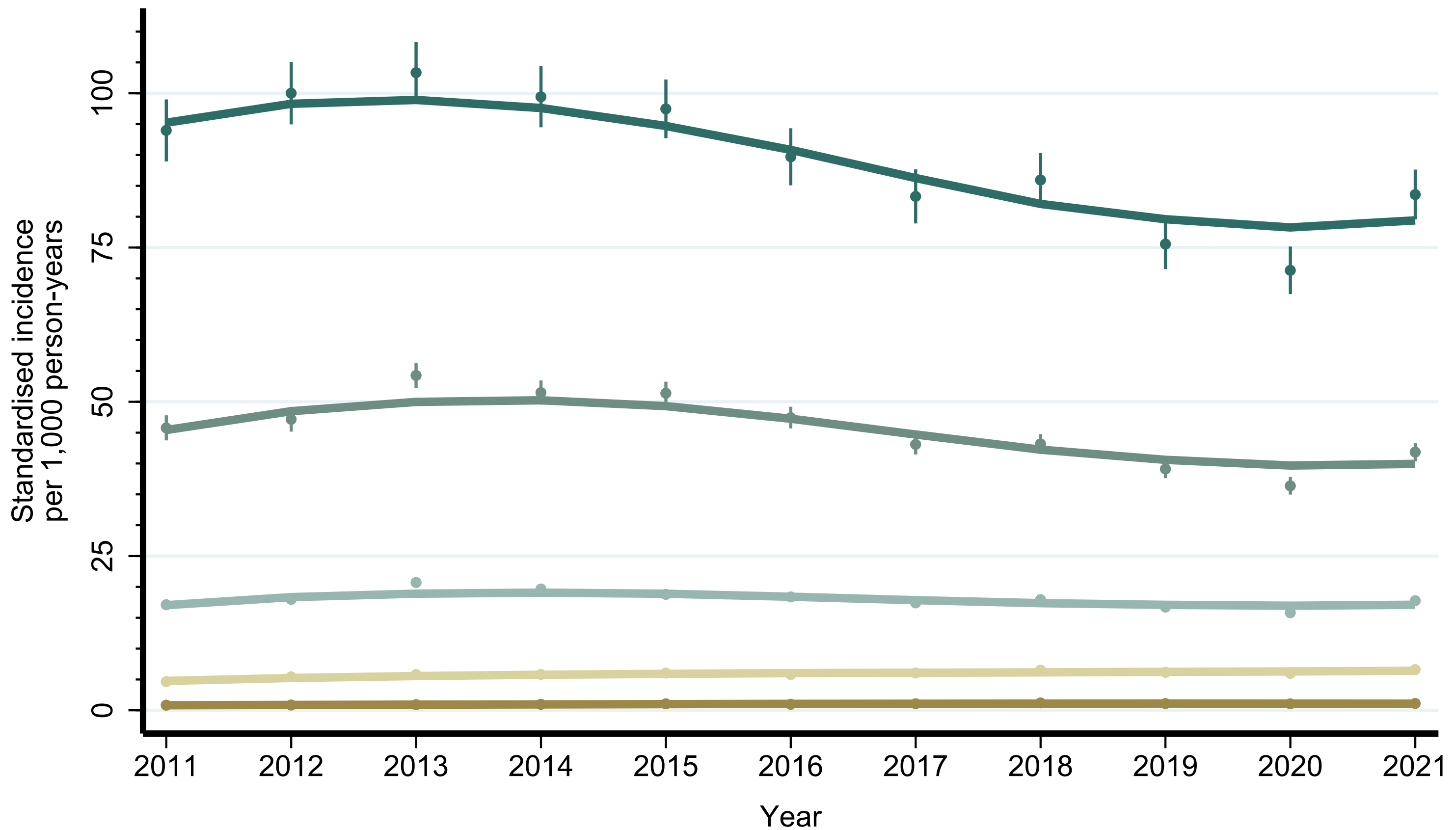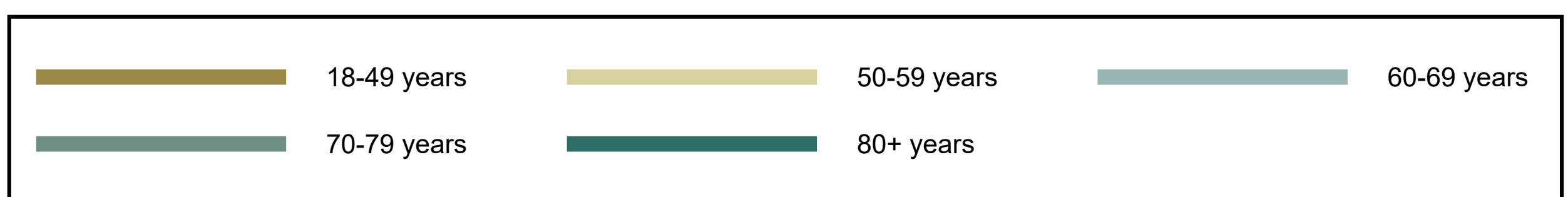

Supplement: sfae351_Supplemental_Files [file sfae351_supplemental_files.zip › Figure S2 - Std. incidence by age groups.pdf]

## Females

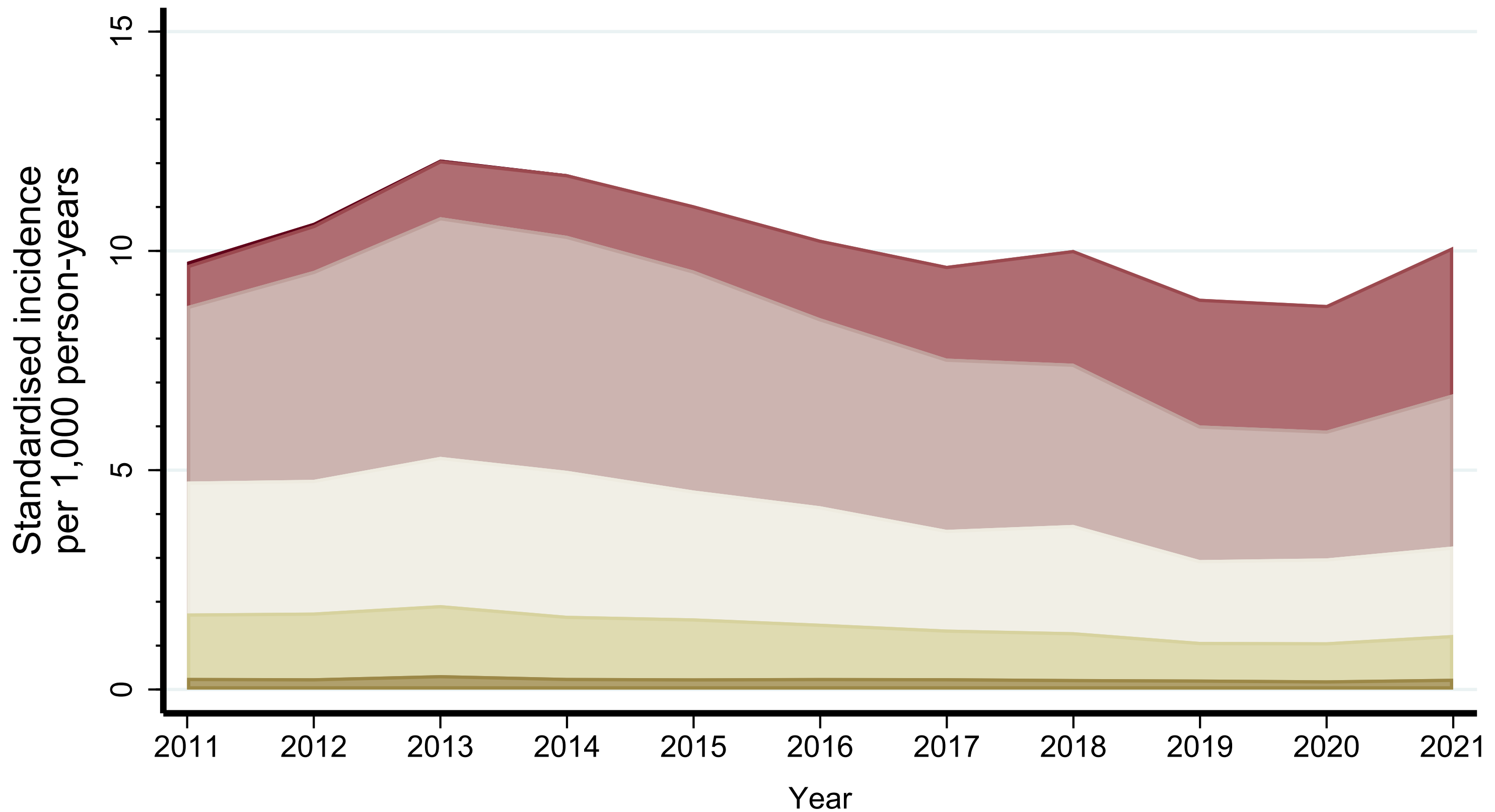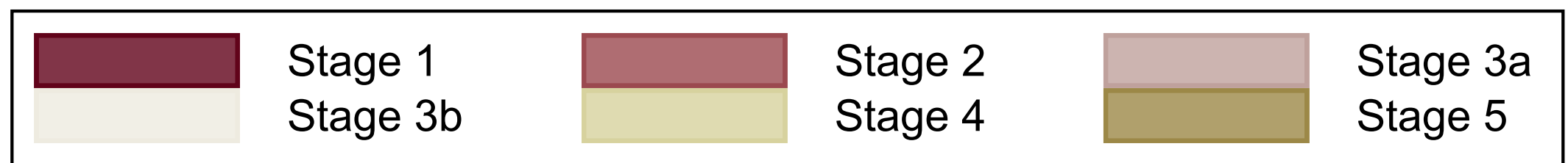

## Males

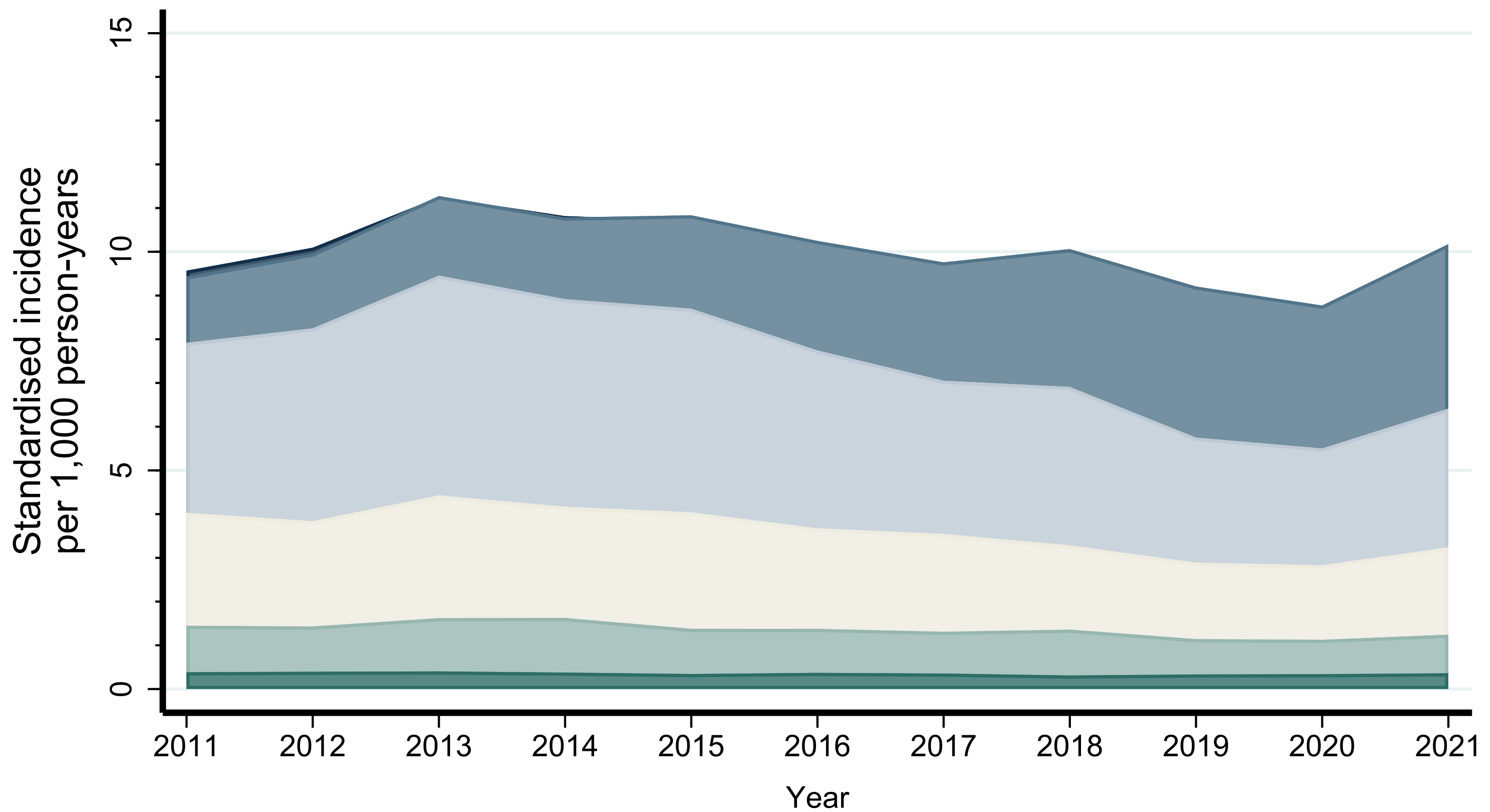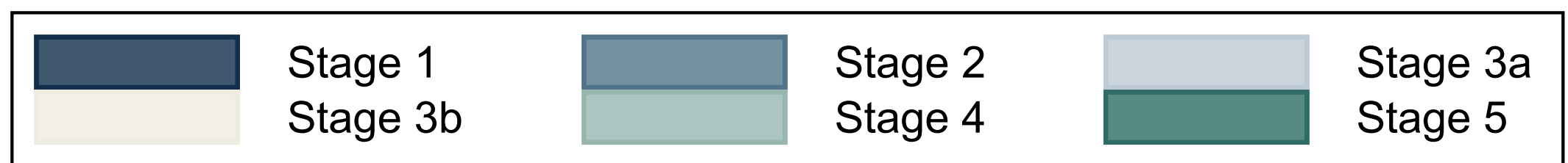

Supplement: sfae351_Supplemental_Files [file sfae351_supplemental_files.zip › Figure S3 - Std. incidence (stages).pdf]

## Females

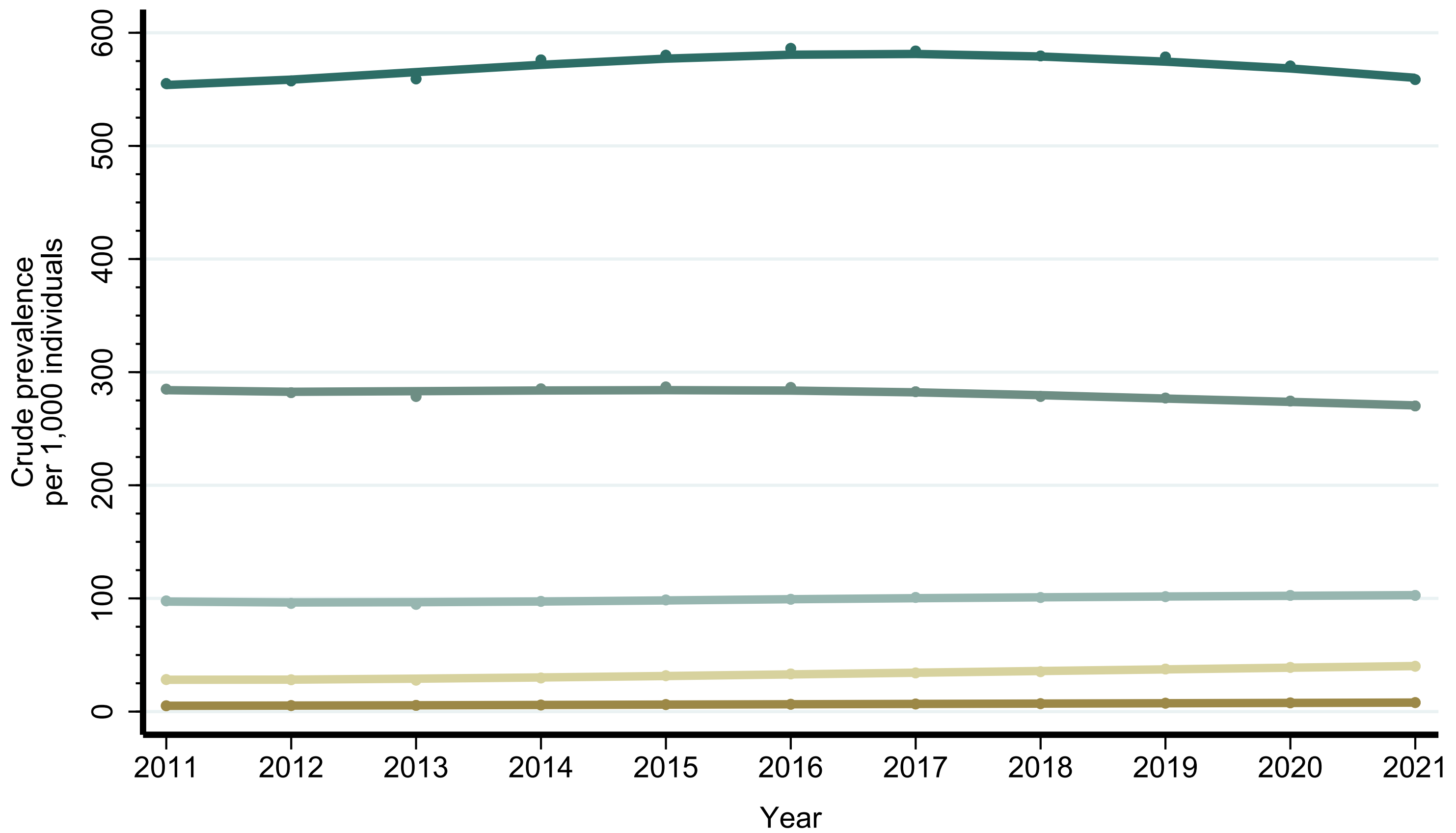

## Males

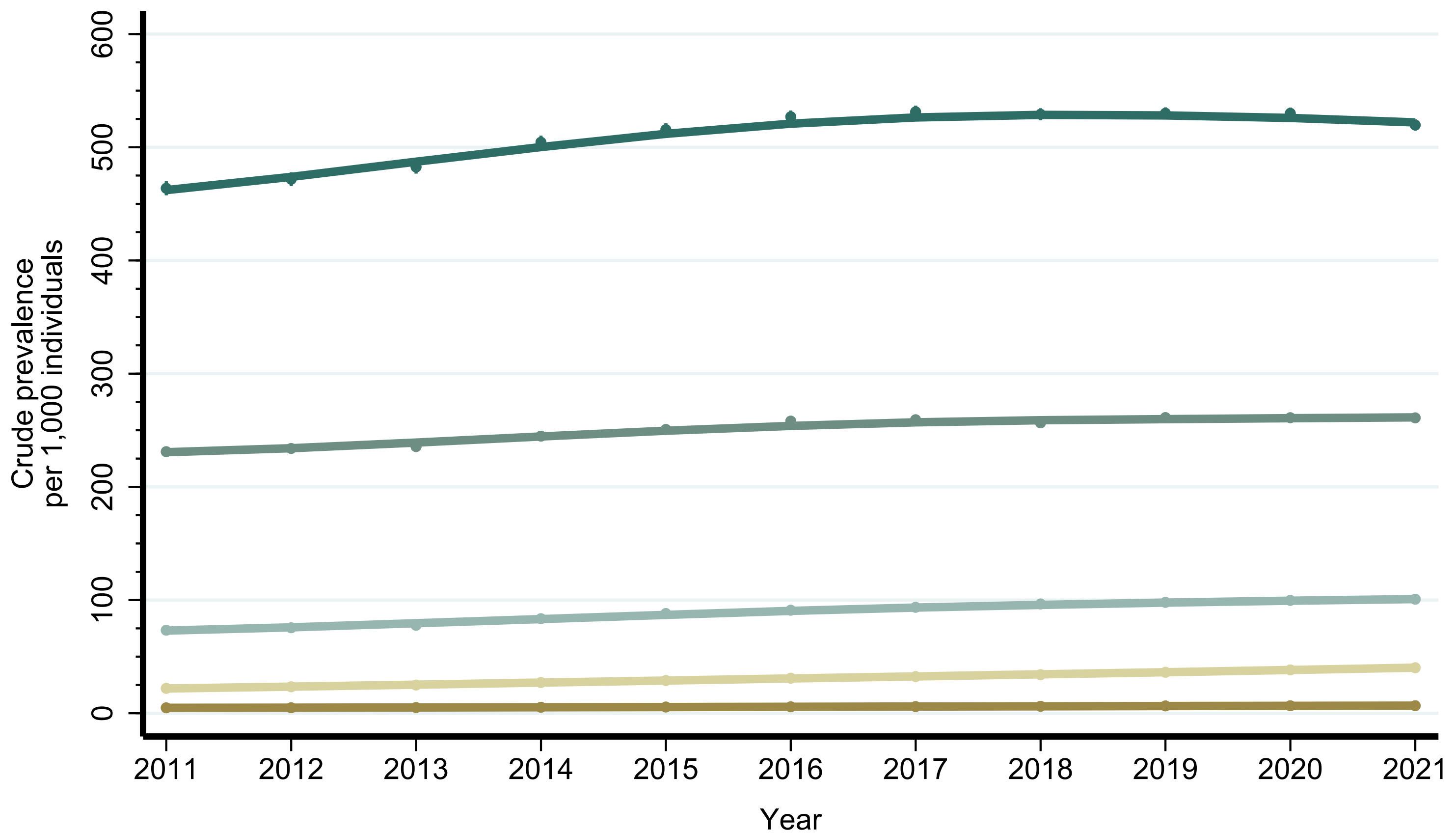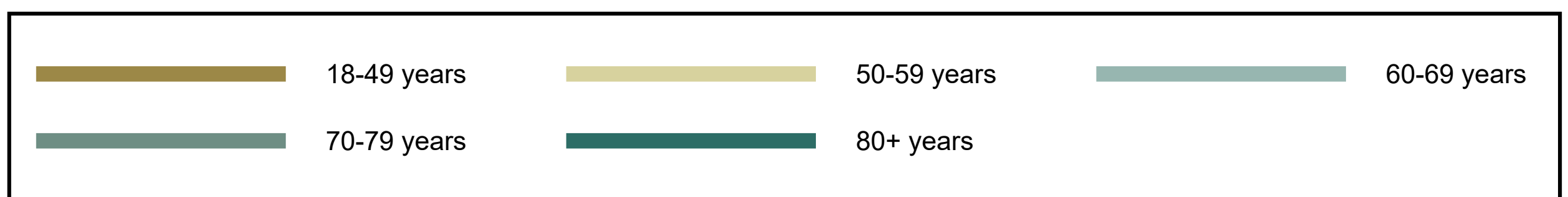

Supplement: sfae351_Supplemental_Files [file sfae351_supplemental_files.zip › Figure S4 - Crude prevalence by age groups.pdf]

## Females

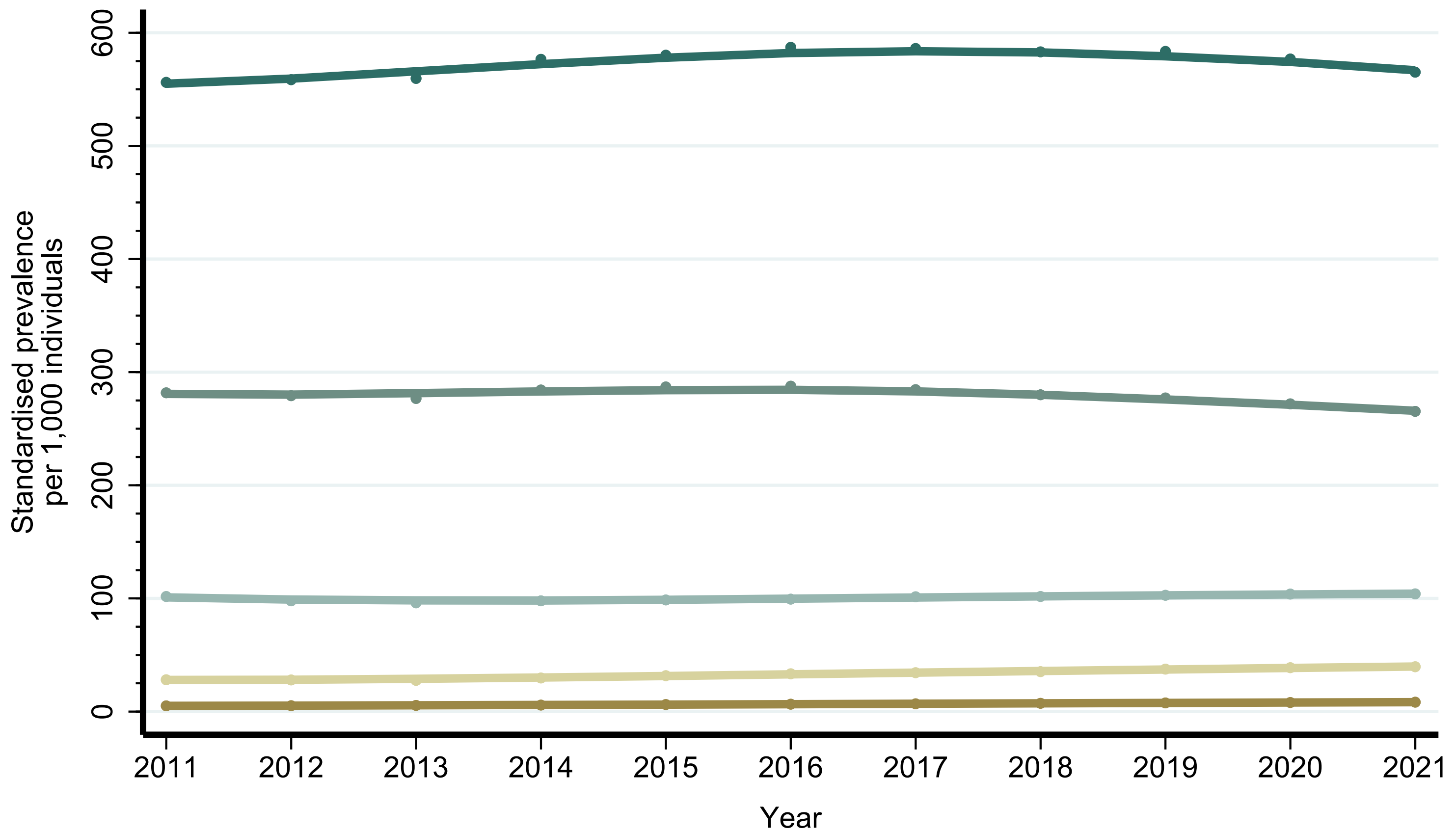

## Males

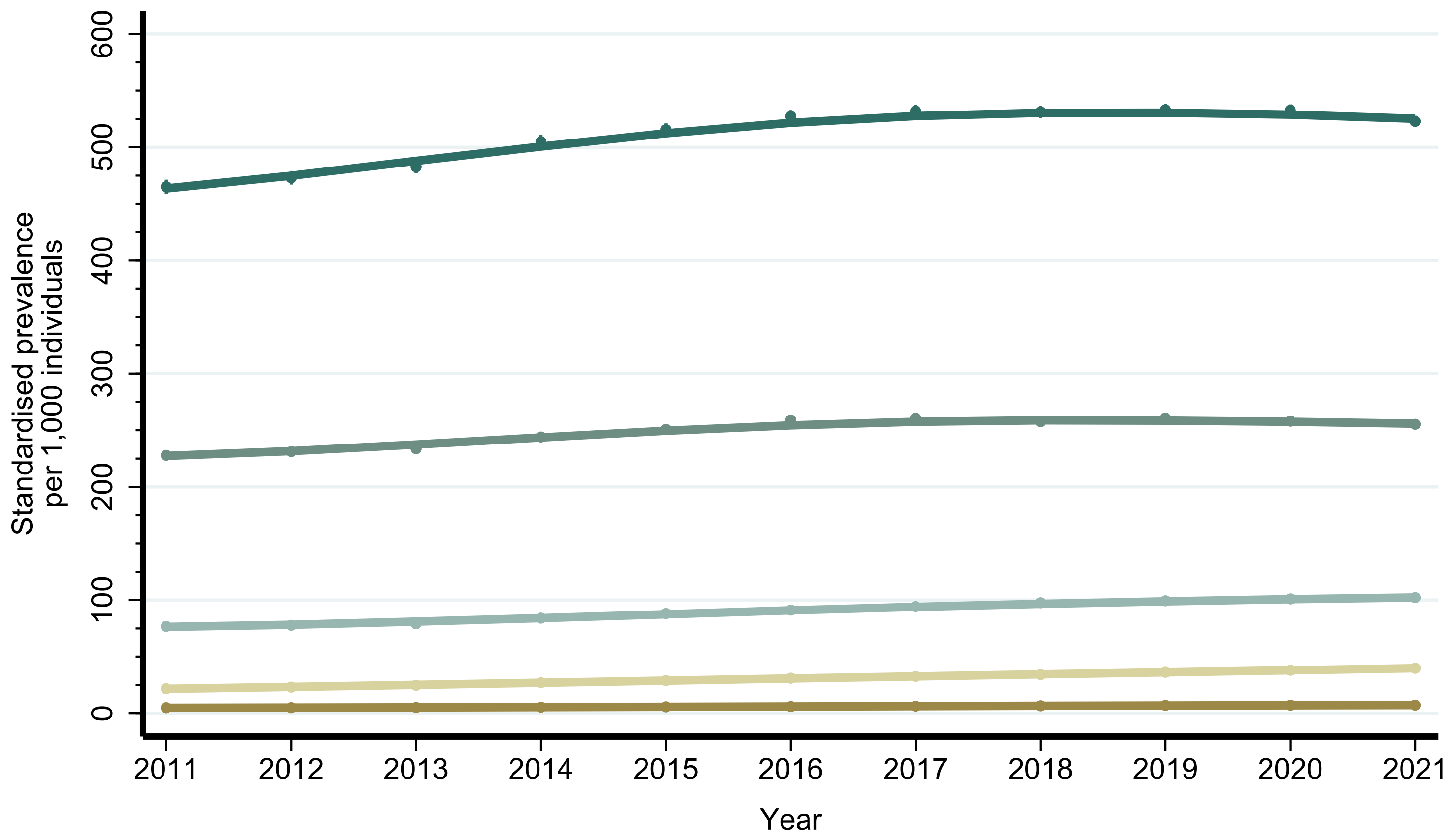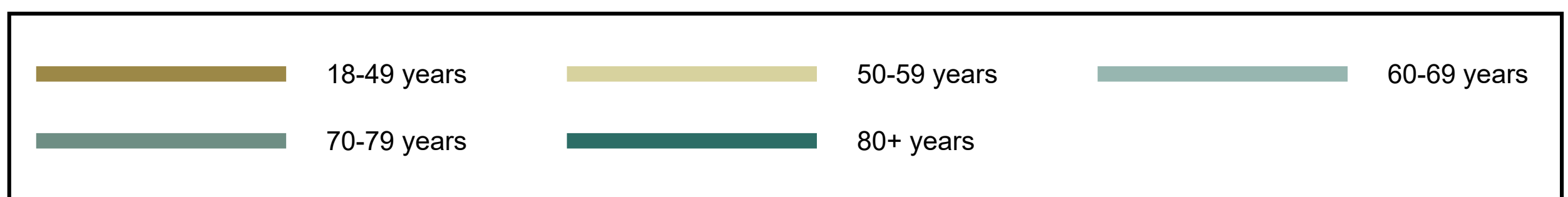

Supplement: sfae351_Supplemental_Files [file sfae351_supplemental_files.zip › Figure S5 - Std. prevalence by age groups.pdf]

## Females

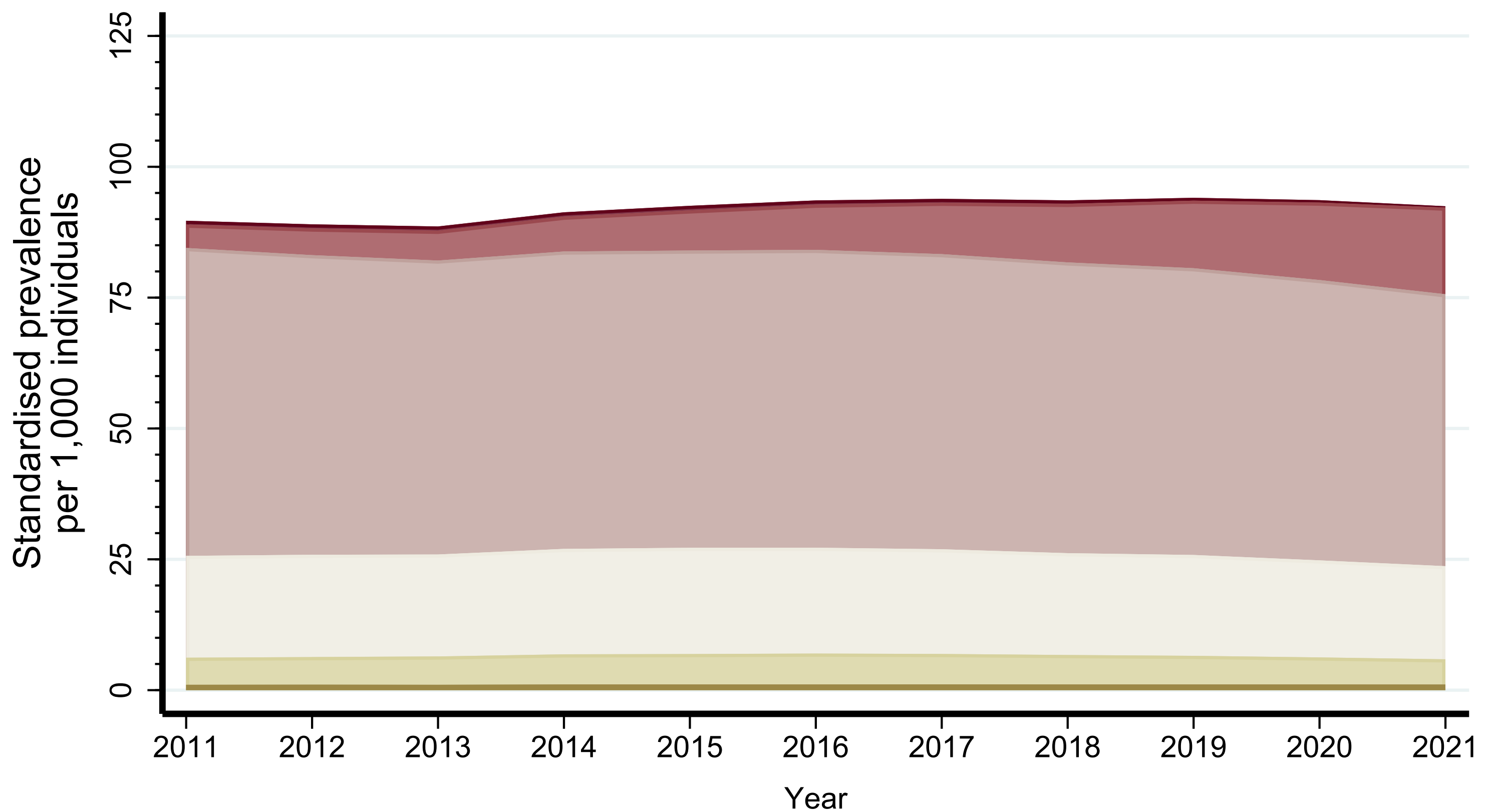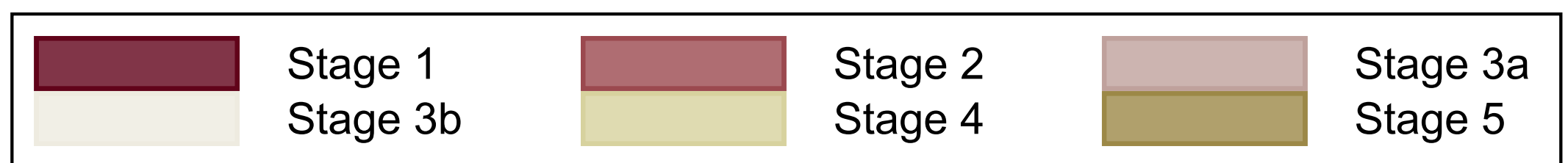

## Males

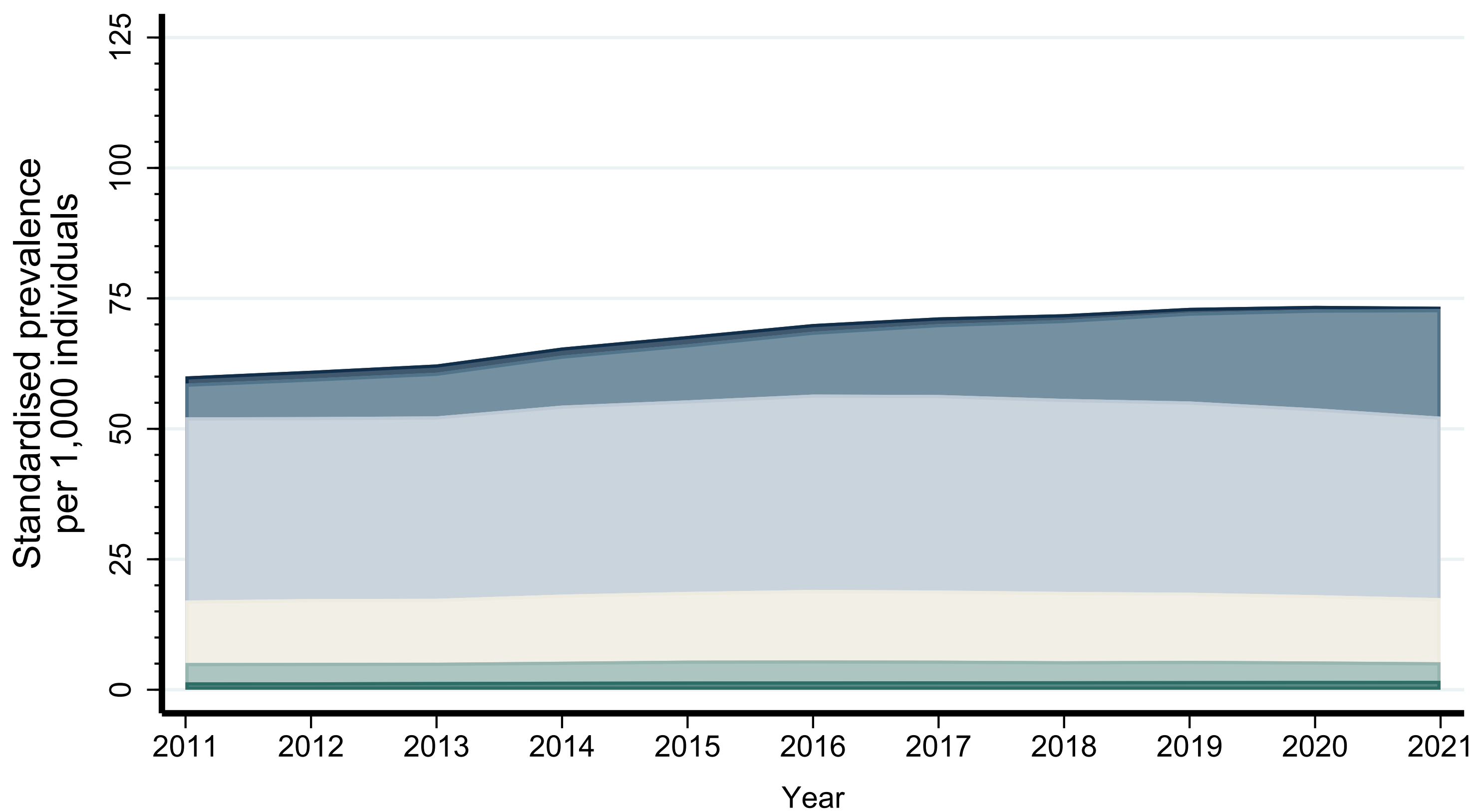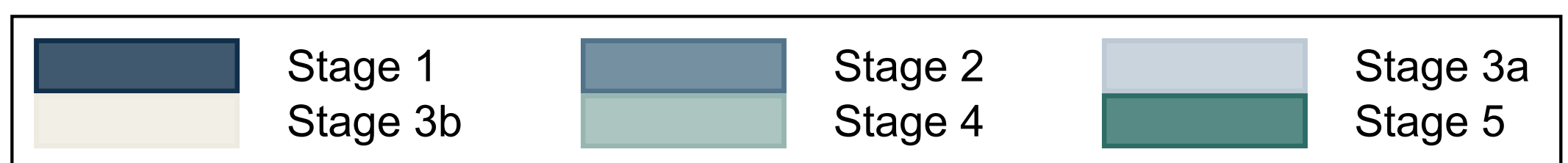

Supplement: sfae351_Supplemental_Files [file sfae351_supplemental_files.zip › Figure S6 - Std. prevalence (stages).pdf]

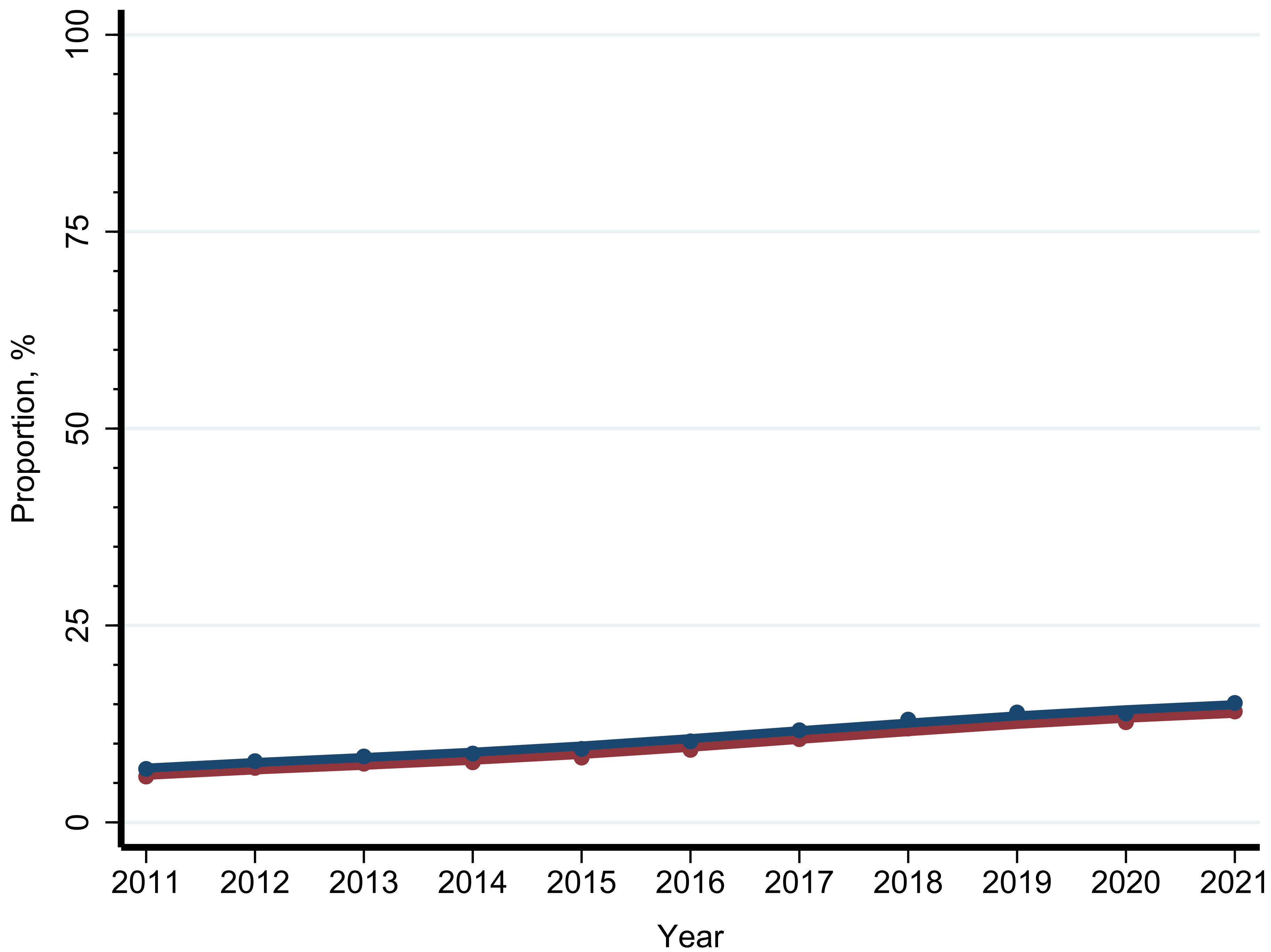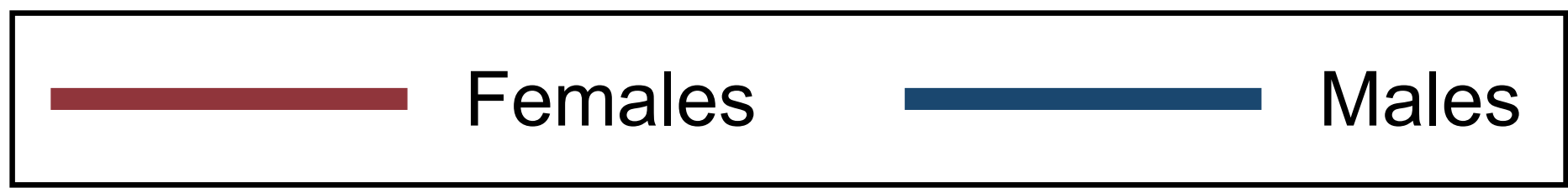

Supplement: sfae351_Supplemental_Files [file sfae351_supplemental_files.zip › Figure S9 - uACR testing.pdf]
